# Exploring the Link Between Mentoring and Intangible Outcomes of Entrepreneurship: The Mediating Role of Self-Efficacy and Moderating Effects of Gender

**DOI:** 10.3389/fpsyg.2020.01556

**Published:** 2020-07-03

**Authors:** Martin Mabunda Baluku, Leonsio Matagi, Kathleen Otto

**Affiliations:** ^1^Department of Educational, Social and Organizational Psychology, School of Psychology, Makerere University, Kampala, Uganda; ^2^Work and Organizational Psychology, Department of Psychology, Philipps-University Marburg, Marburg, Germany

**Keywords:** autonomy, entrepreneurial mentoring, entrepreneurial outcomes, gender differences, intention to stay in self-employment, self-efficacy, work satisfaction

## Abstract

Entrepreneurship education is increasingly becoming a focal strategy for promoting entrepreneurship, particularly to foster entrepreneurial intentions and startups. However, learning and support are equally important after startup for novice entrepreneurs to gain a good level of confidence to manage their business and achieve the desired outcomes. Using a sample of 189 young self-employed individuals in Uganda, this study examines the differential impact of mentoring and self-efficacy on the achievement of intangible outcomes of entrepreneurship including satisfaction of need for autonomy, work satisfaction and the intention to stay in self-employment. We found self-efficacy to mediate the effects of mentoring on these intangible outcomes. In addition, the results showed substantial gender differences. Whereas women’s satisfaction of the need for autonomy and intention to stay in self-employment were strongly associated with the direct effects of mentoring, their male counterparts seemed to benefit more if mentoring resulted in increased self-efficacy. Overall, our findings suggest that whereas mentoring improves the competence of small business owners and consequently achievement of superior outcomes, mentoring should also focus on boosting self-efficacy which in turn is essential for the application of the entrepreneurial competencies.

## Introduction

The need to foster entrepreneurship to boost innovation, self-employment, and economic growth has sparked greater efforts in reviving entrepreneurship education and training (Sánchez, 2013). The assumption is that entrepreneurial training has indirect effects on economic development (Hasan et al., 2017; Nabi et al., 2017; Hahn et al., 2019) through development of the ability to identify and act upon business opportunities (Politis, 2005). While appreciating the contribution of this fast-growing field to entrepreneurship development, it is also important to recognize that it mostly focuses on stimulating new startups. After starting up, entrepreneurs need to continue to learn and receive appropriate support to cope with the challenges of the new business to enable success and persistence. Learning, especially from failure, enables entrepreneurs to gain insights about the critical points in the entrepreneurial process (Cope, 2011). However, entrepreneurs can avoid failure and increase likelihoods of success of new start-ups through continues formal and informal learning from mentors and critical incidents (Sullivan, 2000). The present study, therefore, focuses on entrepreneurial learning through mentoring and its association with entrepreneurs’ perceived level of competence (self-efficacy) and entrepreneurial outcomes.

Entrepreneurial mentoring involves an experienced entrepreneur supporting a prospecting or novice entrepreneur in acquiring the necessary competency for establishing and managing his or her business venture (St-Jean and Audet, 2012; Xiao and North, 2017). The support may appear in different forms including but not limited to experience sharing, role modeling, coaching, apprenticeships, networking, information sharing, motivation, guidance, and feedback (Beckett, 2010; Gong et al., 2011; St-Jean, 2012; Radu Lefebvre and Redien-Collot, 2013; Moore and Wang, 2017). The learning gained from these support efforts and the experiences of the entrepreneur transform into knowledge and skills that enable novices to effectively startup and manage their business ventures (Politis, 2005). Moreover, learning facilitates coping with the challenges of starting and managing a business (Politis, 2005), which may facilitate the attainment of objective and subjective entrepreneurial outcomes including venture performance, entrepreneur satisfaction, and psychological wellbeing. In the present study, we propose that self-efficacy, which partly develops from entrepreneurial learning, mediates the effects of mentoring on entrepreneurial outcomes.

Karlsson and Moberg (2013) claim there is an inadequate understanding of the outcomes of entrepreneurial education. Much of entrepreneurial education and training efforts tend to emphasize the acquisition of cognitive or hard skills. Trainers focus on aspects such as business planning, managing finances, record keeping, savings, and investment. However, effective training and mentoring programs result in the development of non-cognitive skills and resources as well. Notable amounts of affective learning result from entrepreneurial mentoring, which is further associated with benefits relating to the self-concept of the entrepreneur particularly self-efficacy and self-image (St-Jean, 2012). The enhanced perception of an individual’s entrepreneurial abilities through education is associated with behavior (Karlsson and Moberg, 2013), suggesting higher likelihoods of exerting more effort in one’s entrepreneurial activities and consequently higher entrepreneurial success.

In work contexts, mentoring is associated with career clarity, superior performance, adaptability in career and work, job satisfaction, higher income, and professional commitment, (Cascio and Gasker, 2001; Wanberg et al., 2006; Mitchell et al., 2015; O’Mally and Antonelli, 2016). This depicts mentoring as relevant for attaining both objective and subjective work outcomes. In entrepreneurship, mentoring has been associated with objective outcomes specifically skill improvement (Sarri, 2011; Kyrgidou and Petridou, 2013; Gimmon, 2014) which consequently translate into high performance and persistence or business continuity (McKevitt and Marshall, 2015). Our focus is primarily on subjective and intangible outcomes including intrinsic and extrinsic work satisfaction, satisfaction of basic psychological needs (with specific reference to the need for autonomy), and intention to stay in self-employment. In this direction, previous research has demonstrated that mentoring is associated with entrepreneurs’ job satisfaction, and self-efficacy (St-Jean and Audet, 2013; Gimmon, 2014).

In the present study, we highlight the importance of self-efficacy as a mediating mechanism through which entrepreneurial mentoring asserts its influence on satisfaction of need for autonomy, intrinsic and extrinsic work satisfaction, and the desire to stay in self-employment. The study of St-Jean and Mathieu (2015) indicates that self-efficacy mediates the link between mentoring and psychological outcomes such as an entrepreneurial attitude, satisfaction, and persistence. In the present study, we not only test this claim among small business owners in a less developed country but also link mentoring and efficacy to satisfaction of the psychological need for autonomy and the intention to stay in self-employment. Whereas mentoring has numerous benefits to prospecting and novice entrepreneurs, there are variations based on individual differences including gender (Ensher et al., 2000). We, therefore, propose a moderated mediation model such that the effects of mentoring on intangible entrepreneurial outcomes are mediated by self-efficacy and moderated by gender. The theoretical basis for our proposition is presented in the subsequent section.

## Theory and Hypothesis Development

In the present study, we posit that self-efficacy is a mediating mechanism through which entrepreneurial learning accruing from mentoring impacts on entrepreneurial outcomes. Self-efficacy reflects an individual’s belief in his/her abilities and skills to perform a given task and is a precedence for exerting effort, performance, persistence, and success in the task (Bandura, 1997, 2010). Rooted in the social cognitive theory that emphasizes role modeling, person characteristics, and the importance of the environment (Lent et al., 1994, 2002; Lent and Brown, 2013), the self-efficacy theory suggests that self-efficacy develops from mastery experiences, role modeling, social persuasion, and one’s physiological and mood state (Stajkovic and Luthans, 1998; Bandura, 2010). Three of these sources of self-efficacy are reflected in the roles of an entrepreneurship mentor. A mentor is a person who acts as a role model, works together with and encourages the protégée, in addition to providing informational and emotional support, persuading, reassuring, motivating, inspiring, guiding, and integrating the mentee in the entrepreneurship community (St-Jean, 2012; St-Jean and Audet, 2012; Nabi et al., 2019). Working with novice entrepreneurs or offering them apprentice opportunities, role modeling and encouragement are important learning opportunities for the development of entrepreneurial self-efficacy (Wilson et al., 2007).

**FIGURE 1:**
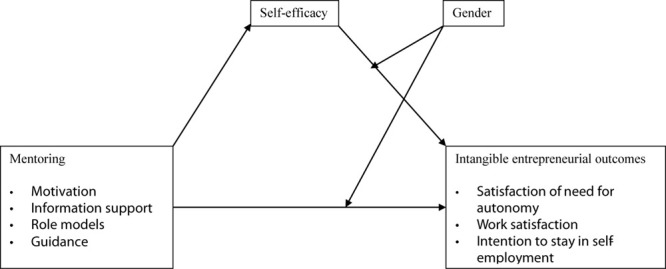
Conceptual model.

One of the central ideas in the self-efficacy theory is that engagement and persistence in a given activity is a function of judgment about one’s skills and capabilities to accomplish the activity but also the ability to cope with the environmental demands in which the activity is conducted (Maddux, 1995). Following this assumption, self-efficacy is associated with shaping thoughts that underlie behavior, regulation of motivation, regulation of emotions, selection of activities and environments (Bandura, 2010). In the entrepreneurial sense, therefore, self-efficacy influences the nature of entrepreneurial activities, the efforts business owners exert in running their ventures, and the affective responses to risks and failures; which further determine the entrepreneurial outcomes. Self-efficacy is an important aspect of perceived behavioral control (Ajzen, 2002) and psychological capital (Luthans et al., 2004, 2015); which constructs are important predictors of engaging in a given behavior. From the psychological capital theory, psychological resources including self-efficacy are associated with commitment, performance, and job satisfaction (e.g., Larson and Luthans, 2006; Luthans et al., 2007b; Avey et al., 2010; Baron et al., 2016a). Hence, self-efficacy could be an important resource for attaining not only the objective entrepreneurial outcomes but also the subjective ones. The predicted associations of self-efficacy with mentoring and intangible entrepreneurial outcomes are indicated in Figure 1 and discussed in the subsequent subsections. However, we begin with elucidating the intangible entrepreneurial outcomes.

### Intangible Outcomes as Measures of Entrepreneurial Success

Individuals go into entrepreneurship for different reasons. Therefore, success does not necessarily have a uniform meaning among all entrepreneurs. To some, it is about creating wealth or financial gain (Parker, 2009). To others, fulfillment of personal goals that are non-financial in nature such as autonomy and independence, self-realization, recognition, and flexible working times may be the expected outcomes (DeTienne et al., 2008; Edelman et al., 2010; Baron et al., 2016a; Baluku et al., 2018b). Even for those who predominantly pursue financial goals, intangible outcomes are also targeted or at least unintended yet vital outcomes. Hence, entrepreneurs’ evaluations of success tend to be more than the objective economic indicators of performance and profits, and therefore important for research to focus on the subjective aspects of success (Baron et al., 2016a; Wach et al., 2016). Subjective entrepreneurial success depicts an individuals’ understanding and evaluation of the valued achievements from the business venture (Dej and Gorgievski, 2012; Wach et al., 2016). In the present study, we focus on specifically subjective and intangible outcomes including satisfaction of the need for autonomy, work satisfaction, and intention to stay in self-employment.

### Satisfaction of Need for Autonomy

The Self-Determination Theory suggests that the autonomous motivation to engage in behavior or activity, which is mostly intrinsic in nature, represents the desire for psychological growth and flourishing (Ryan and Deci, 2000, 2017). Psychological growth, integrity, and wellbeing are attained when three psychological needs including autonomy, competence, and relatedness are satisfied (Ryan and Deci, 2017); hence the pursuit to satisfy these needs is a basis for engaging in activities and behaviors that individuals find inherently interesting (Deci and Ryan, 2000; Ryan and Deci, 2000; García Calvo et al., 2010; Welters et al., 2014). In turn, satisfying these needs facilitates optimum psychological functioning and wellbeing (Deci and Ryan, 2008). In the domain of work, satisfaction of the need for autonomy is particularly regarded as important for workers wellbeing and functioning (Van den Broeck et al., 2010; Otto et al., 2013) and has been cited as one of the major reasons why some people have a preference for an entrepreneurial career (Kolvereid, 1996; Hundley, 2001; van Gelderen, 2010; Croson and Minniti, 2012). It is claimed to be an important determinant of entrepreneurs’ job satisfaction and happiness (Binder and Coad, 2013; Berglund et al., 2015; Baluku et al., 2018b). This psychological need represents the desire for self-regulation, which is different from independence or self-reliance, and rather encompasses behaviors that are congruent to one’s inherent interests and values (Ryan and Deci, 2017). In this paper, we demonstrate how mentoring has the potential for enabling novice entrepreneurs to achieve gratification of the need for autonomy.

### Work Satisfaction

The Industrial and Organizational psychology literature is not devoid of work or job satisfaction research, most of this research is concerned with the intrinsic and extrinsic job satisfaction of employees in organizations though, while rarely job satisfaction of own-account workers or entrepreneurs is taken into account. In the present study, satisfaction denotes the conceptualization of work satisfaction as a state of emotional pleasure accruing from the appraisal of an individual’s work as facilitating the achievement of one’s work values (Locke, 1969). This involves an evaluation of whether one is achieving the intrinsic and extrinsic goals he or she expects from his or her work. Hence, work satisfaction portrays happiness with one’s work; which is often reflected in pleasant moods, emotions, wellbeing and positive attitudes (Fisher, 2010). Similar to satisfaction of employees, both intrinsic and extrinsic aspects of work satisfaction must be considered in self-employment, since these are distinct and may be related differently to other predictor or outcome variables (Hauber and Bruininks, 1986; Hirschfeld, 2000). Work satisfaction as an important work attitude has an influence on several domains of an individual’s life. Therefore, perceptions of satisfaction or happiness at work are extremely important for an individual’s overall happiness (Olsson et al., 2013; De Neve and Ward, 2017). In the entrepreneurial context, work satisfaction has a spillover effect on other entrepreneurial outcomes including venture performance and profits (Dijkhuizen et al., 2016) and willingness to persist in an entrepreneurial role (Baluku et al., 2018a). Whereas entrepreneurs job satisfaction has previously been linked to individual attributes such as personality (Berglund et al., 2015), work-person fit (De Jager et al., 2016; Langer et al., 2019) and attainment of work autonomy (Sappleton and Lourenço, 2016; Baluku et al., 2018b; Shir et al., 2018), we posit in the present paper that mentoring and the resulting self-efficacy are also foundations for achieving satisfaction in entrepreneurship work.

### Intention to Stay in Self-Employment

The willingness to continue with work in the entrepreneurship field could be an important proxy indicator of the positive evaluation of their work and outcomes. Extant research has investigated the related constructs of entrepreneurial success, failure, exit, and re-entry. However, literature is silent on entrepreneurs’ intention to stay in their roles for a long time. Patel and Thatcher (2014) labeled this phenomenon as persistence in self-employment, while other researchers have investigated it in terms of commitment to one’s own business (Felfe et al., 2008; Baluku et al., 2018a, b,; Schummer et al., 2019). Persistence in entrepreneurial work is important for realization of the economic benefits of entrepreneurship since these tend to accrue in the long term than in the short term (Baluku et al., 2018a; Schummer et al., 2019). Having the intention to stay in this form of employment, which reflects the commitment to the form of employment (Felfe et al., 2008) generates higher morale and effort, hence an essential attitude that can stimulate attainment of other work outcomes (Felfe et al., 2008).

### Entrepreneurial Mentoring

Scholars and practitioners alike are increasingly focusing attention on entrepreneurship education. The assumption is that entrepreneurial learning has the potential to stimulate successful innovations and entrepreneurial startups through the acquisition of entrepreneurial competencies, development of positive entrepreneurial attitudes, and fostering innovative ideas (Man, 2019; Wang et al., 2019; Wei et al., 2019). Whereas much attention is being paid to entrepreneurship education in universities and other formal settings, learning that supports entrepreneurship development in informal settings should not be forgotten or ignored. Moreover, the effectiveness of entrepreneurial mentoring could be dependent on the context (Ting et al., 2017). It has been posited that informal mentoring is well suited to small business owners given the context in which they operate (McKevitt and Marshall, 2015). In this study, we particularly focus on the impact of informal mentoring on attaining intangible entrepreneurial outcomes.

At the general level, entrepreneurial mentoring is a learning process in which the experienced entrepreneur supports the development of a prospecting or novice entrepreneur (Beckett, 2010; Gong et al., 2011; St-Jean and Audet, 2012; Xiao and North, 2017). Entrepreneurial mentoring facilitates entrepreneurial learning in a number of ways including motivation, information support, counseling, reflection, integration, guidance, and role modeling (St-Jean, 2012). Like formal mentoring relationships, informal mentoring is important and has the potential for stimulating attainment of important entrepreneurial outcomes including persistence and survival, reduction in costs, satisfaction, psychological wellbeing, and business leadership. Evidence from research on informal and formal mentoring among organizational employees shows that informal mentoring may actually have stronger positive impact on self-efficacy and leadership as well as on work outcomes such as salary, intrinsic job satisfaction, and commitment (Chao et al., 1992; Raabe and Beehr, 2003; Van Emmerik, 2004). This is because they report receiving better support than those in formal mentoring situations (Chao et al., 1992). Mentoring functions such as information sharing, support with creating networks, guidance and experience sharing (Beckett, 2010; Gong et al., 2011; Radu Lefebvre and Redien-Collot, 2013) frequently occur informally especially among small business owners in the informal sector. Such mentoring can come from entrepreneurial socializing agents including family, peers, and friends who are experienced in business, role models, and others who support skill development and provide essential resources including information and knowledge. All these aid novice entrepreneurs to adjust to their entrepreneurial roles (Starr and Fondas, 1992; Krueger, 2007). To highlight the role of informal mentoring, Brodie et al. (2017) suggest that formal mentoring programs should be supplemented by informal mentoring relationships in form of peer support; and where possible both formal and informal mentoring need to be incorporated in entrepreneurial learning programs (Edwards and Muir, 2005).

Like formal mentoring, informal mentoring facilitates skills acquisition and change in attitudes among prospecting and novice entrepreneurs (Ahmed et al., 2017; Baluku et al., 2019b), hence improves competence for opportunity recognition and efficacy for action. Mentors in the informal setting tend to offer more hands-on training and practical information since this type of learning occurs in the natural business environment and on the job. Moreover, practical training is associated with superior entrepreneurship learning outcomes (Autio et al., 2001; Fayolle and Gailly, 2015; Huq and Gilbert, 2017).

The immediate outcomes of entrepreneurship education and learning include the acquisition of entrepreneurial skills and knowledge. Beyond professional skills such as business planning and financial management, mentoring does support the development of soft and affective skills that are related to the core functions of an entrepreneur. Soft skills such as self-efficacy or boost in confidence are important (St-Jean and Audet, 2012; Brodie et al., 2017) which further facilitate the application of entrepreneurial skills. In addition, entrepreneurial mentoring offers novice entrepreneurs a platform for obtaining emotional support, learning to make decisions, building a professional identity and belonging to the entrepreneurship community (Terjesen and Sullivan, 2011; St-Jean and Audet, 2012; Radu Lefebvre and Redien-Collot, 2013). These, in turn, should facilitate the achievement of intangible entrepreneurial outcomes including wellbeing, autonomy, satisfaction, and the desire to persist in the entrepreneurial role. Concerning the dimensionality of work satisfaction, although extant research has mainly focused on intrinsic aspects, there is evidence suggesting that mentoring also has positive effects on extrinsic satisfaction of individuals small businesses (e.g., Lo and Ramayah, 2011). Both formal and informal support, guidance, and other mentoring functions have the potential to facilitate creation of better working conditions for one’s self or appreciation of the work environment, dealing with one’s employees and taking decisions in a better way, thereby enhancing extrinsic satisfaction. Considering the above literature, we hypothesize that:

Hypothesis 1: Entrepreneurial mentoring is positively related to the self-efficacy of entrepreneurs.
Hypothesis 2: Entrepreneurial mentoring positively predicts (a) satisfaction of the need for autonomy (b) intrinsic work satisfaction, (c) extrinsic work satisfaction, and the (d) intention to stay in self-employment.

### The Role of Self-Efficacy

It has been suggested that prospecting entrepreneurs should be supported to develop their self-efficacy given their limited experience and knowledge of the entrepreneurial process (Ahsan et al., 2018). Mentoring not only supports them to develop the entrepreneurial self-efficacy but also facilitating the process of transiting into entrepreneurship and building their identity as entrepreneurs (Ahsan et al., 2018; Newbery et al., 2018). In line with the Social Cognitive Career Theory (Hackett and Lent, 1992; Lent and Brown, 2013), entrepreneurial self-efficacy develops from entrepreneurial socialization or learning occurring through education, training, and experiences that enhance the skills and mastery experiences of novice entrepreneurs. In a number of studies, individuals who have undertaken entrepreneurial training formally or informally have reported higher levels of entrepreneurial self-efficacy (Ho et al., 2018; St-Jean and Tremblay, 2020). However, this may be dependent on the protégés’ learning orientation (St-Jean et al., 2018). Nonetheless, self-efficacy is in turn associated with career outcomes including commitment to goal-directed behavior, performance, satisfaction and wellbeing (Lent and Brown, 2008, 2013). In the entrepreneurship field, self-efficacy determines several entrepreneurship behaviors and outcomes including creativity, innovativeness, and performance (McGee and Peterson, 2019). We posit that self-efficacy not only determines attainment of objective outcomes of entrepreneurship, but also the subjective and intangible outcomes in different ways.

Self-efficacy, also referred to as confidence (Luthans, 2002; Luthans et al., 2015) is the subjective evaluation of one’s own abilities to perform a specific task in a given context (Bandura, 1997; Luthans and Peterson, 2002). It includes mobilizing cognitive resources, motivation, and taking required steps in executing the given task (Stajkovic and Luthans, 1998; Luthans and Peterson, 2002; Luthans et al., 2007c). People are not only attracted to but also achieve more in activities or careers where their efficacy is higher (Forbes, 2005).

Although some researchers have demonstrated that high levels of entrepreneurial self-efficacy harms business success (Jain and Ali, 2013; Artinger and Powell, 2016; Baron et al., 2016b), entrepreneurship is highly challenging and risky and hence requires sufficient amounts of psychological resources (Baron et al., 2016a). As a psychological resource, self-efficacy is useful in recognizing opportunities and soliciting resources for a start-up (Boyd and Vozikis, 1994; Dimov, 2010; Culbertson et al., 2011). Previous research has also shown that self-efficacy is related to lower fear of failure and reduced risk perception (Krueger and Dickson, 1994; Goel and Karri, 2006) and boosts likelihoods of persistence (Cardon and Kirk, 2015). Persistence in challenging activities or careers is mainly a function of self-efficacy (Goel and Karri, 2006; Dimov, 2010; Bullough et al., 2014).

Self-efficacy also enhances entrepreneurial outcomes through its usefulness in resolving conflicts with stakeholders to the business (Zou et al., 2016), business leadership, decision making, and risk management (Kuratko, 2007; Mattare, 2008). Moreover, self-efficacy tends to boost job satisfaction and wellbeing (Karademas, 2006; Duggleby et al., 2009; Skaalvik and Skaalvik, 2014). Self-efficacy is associated with work success (Judge et al., 2001; Luthans et al., 2006), both intrinsically and extrinsically which consequently translates into extrinsic and intrinsic work satisfaction. Self-efficacy further boosts the intrinsic aspect through its role in facilitating persistence and dealing with difficulties (Yakın and Erdil, 2012), which are important in entrepreneurship. The confidence boost arising from entrepreneurial mentoring should not only translate into performance, but also the ability to make independent decisions and undertake autonomous action, i.e., satisfying the need for autonomy, but also satisfaction with one’s work and the desire to continue working in the entrepreneurship role. Previous research has already indicated that entrepreneurial mentoring impacts the satisfaction and retention of novice entrepreneurs through self-efficacy (St-Jean and Mathieu, 2015). In the present study, we examine these claims in the context of a less developed country, and also focus on more intangible outcomes.

Hypothesis 3: Self-efficacy mediates the relationship between entrepreneurial mentoring and (a) satisfaction of the need for autonomy (b) intrinsic work satisfaction, (c) extrinsic work satisfaction, and (d) intention to stay in self-employment.

### Gender Differences

There is a big gender gap in entrepreneurship (Guzman and Kacperczyk, 2019). This is not only true regarding the number of women going into entrepreneurship but also in terms of success and persistence (Smith and Tolbert, 2018; Oppedal and Garcia, 2019) and is more pronounced in high-growth ventures (Scott and Shu, 2017). Moreover, even efforts to improve entrepreneurial outcomes tend to be more successful among men than women due to several constraints including time and low credit (Oppedal and Garcia, 2019). This could limit the possible positive effects of entrepreneurial mentoring and self-efficacy on women’s entrepreneurial success. This may not be limited to entrepreneurial situations only, as students’ perceptions of mentoring generally seem to be gendered, with females seeking more of psychological and emotional support (Deale et al., 2020).

The business environment is certainly gendered, both culturally and socially (Bruni et al., 2005), with males dominating entrepreneurial platforms. In this direction, Marlow and McAdam (2013) argue that even reports of underperformance of female-owned enterprises represent a gender bias in entrepreneurship debates, given that small enterprises tend to have low performance levels. Given these dynamics, we investigate whether men and women benefit from mentoring equally, in relation to the realization of intangible entrepreneurial outcomes. Contrary to the idea that women are underprivileged in the business environment, women tend to have a higher drive to succeed and persist in business given the opportunity offers it for work-family balance (Baron and Henry, 2011). Moreover, entrepreneurship has been found to enhance women’s empowerment, self-drive, and autonomy (Apitzsch, 2003; Datta and Gailey, 2012; Zgheib, 2018). Consequently, women may report higher work satisfaction and need to stay in self-employment.

It has been observed that low self-efficacy is one of the barriers to women’s engagement in more lucrative business industries (Wieland et al., 2019). However, when women in mentoring programs are fully committed to their ideas, they are likely to achieve similar results as their male counterparts especially in terms of venture financing and commercialization (Scott and Shu, 2017). Although this shows that men and women could benefit equally from entrepreneurial mentoring, it is more possible if all factors such as gender roles, access to resources, and social cultural constraints are kept constant for both males and females. In contexts where gender roles are emphasized, for example the orientation of males toward competition, men are more likely to benefit from entrepreneurial mentoring (Bergman et al., 2011). Moreover, in formal mentoring, it seems only women with higher confidence tend to go into entrepreneurial mentoring programs (Bergman et al., 2011). The situation could even be more skewed in favor of men in informal mentoring, given that entrepreneurial spaces are dominated by men. Consequently, there fewer female role models and mentors that prospecting or nascent female entrepreneurs can learn from. On the positive side, it has been demonstrated that self-efficacy has stronger effect on girls’ entrepreneurship interest (Kickul et al., 2008). In addition, it remains questionable if women receiving the same level of mentoring and perceiving a comparable amount of self-efficacy as men report the same level of entrepreneurial outcomes? In this regard, we hypothesize that:

Hypothesis 4: The direct effects of entrepreneurial mentoring on (a) satisfaction of the need for autonomy (b) intrinsic work satisfaction, (c) extrinsic work satisfaction, and (d) intention to stay in self-employment are stronger for men than for women.
Hypothesis 5: The indirect effects of entrepreneurial mentoring on (a) satisfaction of the need for autonomy (b) intrinsic work satisfaction, (c) extrinsic work satisfaction, and (d) intention to stay in self-employment via self-efficacy are stronger for men than for women.

## Materials and Methods

### Participants and Procedure

The sample comprised of 188 (86 females, 102 males) young business owners in Uganda’s capital, Kampala. These were young people who had recently graduated from high school, technical/vocational colleges, and universities; and are engaged in self-employment. Participants were recruited through youths’ business forums, while others were approached at their business premises and requested to participate in the survey. The survey questionnaires were administered through the paper and pencil method. Participants were aged 17 to 30 years (*M* = 24.72, *SD* = 7.99). Given that participants were young and recently graduated from school, their businesses were nascent. The average time participants had spent in business was 2.58 years (*SD* = 0.99) with only 3.19% reporting that they have been in business for 5 or more years. Most of the participants were graduates of universities or technical colleges, with 51.6% being degree holders and 22.34% having ordinary and advanced certificates in technical or vocational studies. It was also observed that 40.43% had studied business related courses.

### Measures

***Mentoring*** was measured using the entrepreneurial mentoring questionnaire in Baluku et al. (2019a). Only 10 most valid items assessing the level to which an individual has had access to different aspects of entrepreneurial mentoring during the last year on a 5-point Likert type scale; 1 (never) to 5 (always). The items included (1) Someone has encouraged to discuss how I feel about ability to succeed in self-employment; (2) Someone has encouraged to discuss with him or her my honest feelings and business experiences; (3) Someone has helped me to explore realistic ways for achieving my business objectives; (4) I have been provided with practical suggestions for succeeding in business; (5) Someone has expressed his or her own confidence in my ability to succeed in business; (6) Someone has used his or her own personal experience to explain how I can achieve career and financial success in business; (7) Someone has guided me to explore my personal strengths that can be useful to doing business; (8) In interactions with mentors and role models, I have been offered recommendations on how to improve my business acumen; (9) I have been guided on how to assess business opportunities; and (10) I have had help developing better coping strategies when I have not achieved my business goals. These items showed high internal consistency (α = 0.93).

To measure ***Self-Efficacy***, we adapted items from the Psychological Capital Questionnaire – (Luthans et al., 2007a). Participants indicated their degree of agreement with three statements (I feel confident in analyzing the problems of business to find solutions; I feel confident in presenting my business and ideas in different business forum; I feel confident presenting information to a group of business colleagues). The items were rated on a 6-point Likert scale ranging from 1 (strongly disagree) to 6 (strongly disagree), and showed an acceptable level of internal consistency (α = 0.74).

To measure ***Satisfaction of the Need for Autonomy***, we adopted the short measure from Deci and Ryan’s Basic Psychological Needs Scale (Samman, 2007; 464–465). The scale is comprised of three items measured on a 4-point scale from 1 (not at all true) to 4 (completely true). A sample item is “I feel like I can pretty much be myself in daily situations.” The reliability of this scale in the present study was α = 0.78.

*Work Satisfaction* was measured using items from the revised short form of the Minnesota satisfaction questionnaire (Hirschfeld, 2000). Participants were asked to indicate the level of satisfaction with the different aspects of their work. We measured both intrinsic and extrinsic aspects of work satisfaction. Intrinsic work satisfaction was measured with items 7, 9, 11, 15, 16, and 20; while extrinsic work satisfaction was measured with items 5, 6, 8, and 17. The remaining items were dropped because of low loading during factor analysis. Sample items are “the feeling of accomplishment I get from the job” for intrinsic satisfaction; and “The way my job provides for steady employment” for extrinsic satisfaction. The items were measured on a 5-point Likert scale ranging from 1 (strongly disagree) to 5 (strongly agree). The items showed a good level of internal consistency at α = 0.77 for both intrinsic and extrinsic work satisfaction.

To measure the ***Intention to Stay in Self-Employment***, we adapted four (4) of the six (6) items from the career commitment scale (Blau, 1985, 1988). The scale measures an individual’s level of commitment or readiness to change his/her occupation. In the present study, we adapted the scale to measure commitment to continue in the self-employment occupation. The adapted items include (1) I want to make a long career in self-employment; (2) If I had all the money needed, I would still want to be self-employed; (3) I like my career in self-employment too well to give it up; and (4) Self-employment is ideal vocation for a life work. These items were measured on a 5-point Likert scale ranging from 1 (strongly disagree) to 5 (strongly agree) and showed a good level of internal consistency at α = 0.89.

### Analytic Strategy

We used the PROCESS macro version 3.4 (Nathan and Scobell, 2012) to test our hypotheses. We applied model 15 of the PROCESS macro, which computes for the moderation mediation effects simultaneously. Hence, entrepreneurial mentoring was entered as the focal predictor, self-efficacy as the mediator, gender (Female = 0, Male = 1) as the moderator. We computed a different model for each outcome variable (i.e., satisfaction of need for autonomy, work satisfaction, and intention to stay in self-employment). In each regression model, we controlled for the effects of age because it tends to affect entrepreneurial outcomes, and particularly psychological outcomes such as wellbeing (Baron et al., 2016a). In addition, we applied sample bootstrapping at 5,000 in line with Hayes’ (2013) recommendation. Common methods bias is one of the challenges in behavioral surveys that might arise from item characteristic effects, item context effects, and measurement context (Podsakoff et al., 2003). To rule out the common methods bias concern for our study, we used Harman’s single factor test and total variance of the single factor was 35%, suggesting that the variance in the variables was accounted for by several factors. Hence, common methods bias was not a concern for this study. However, this method has been criticized as insufficient (Podsakoff et al., 2003; Chang et al., 2010). In addition, the variance inflation factors ranged from 1.06 to 1.62 which are within the acceptable limits (Hair et al., 2011), hence our data had no collinearity concerns to worry about despite the high correlations between some of the variables.

## Results

Correlations among study variables and descriptive statistics are presented in Table 1. The moderated mediation regression results are reported in Table 2. The findings showed support for our first hypothesis that entrepreneurial mentoring is positively associated with entrepreneurs’ self-efficacy (*B* = 0.46, *p* < 001). In addition, the control variable (age) was positively related to self-efficacy (*B* = 0.17, *p* < 01) but not with the intangible entrepreneurial outcomes. The moderator variable (gender) had substantial effects on all three outcomes: satisfaction of the need for autonomy (*B* = 0.15, *p* < 05), intrinsic work satisfaction (*B* = −0.47, *p* < 001), extrinsic work satisfaction (*B* = −0.61, *p* < 001) and the intention to stay in self-employment (*B* = 0.25, *p* < 01). The negative association between gender and work satisfaction implies that women were more satisfied with their work than their male counterparts.

**TABLE 1:** Descriptive statistics and correlations among study variables.

Variables	Mean	(Min, Max.)	SD	1	2	3	4	5	6	7
**Sex**										
Entrepreneurial mentoring	3.13	(1, 5)	0.99	0.16*	(0.94)					
Self-efficacy	4.16	(1, 6)	0.75	0.28***	0.55***	(0.74)				
Satisfaction of need for autonomy	2.72	(1, 4)	0.76	0.29***	0.63***	0.64***	(0.78)			
Intrinsic work satisfaction	3.77	(1, 5)	0.65	−0.28***	0.35***	0.32***	0.22**	(0.77)		
Extrinsic work satisfaction	3.73	(1, 5)	0.75	−0.30***	0.18*	0.34***	0.22**	0.75***	(0.77)	
Intention to stay in SE	2.97	(1, 5)	0.98	0.20**	0.61***	0.55***	0.61***	0.35***	0.20**	(0.89)

* *p* < 0.05, ** *p* < 0.01, *** *p* < 0.001. Gender (Female = 1, Male = 0). Cronbach’s α in diagonal parenthesis.

**TABLE 2:** Moderated mediation effects of mentoring on intangible entrepreneurial outcomes.

	Self-efficacy			Satisfaction of need for autonomy			Intrinsic work satisfaction			Extrinsic work satisfaction			Intention to stay in self-employment		
	*B*	*SE*	*CI(LL, UL)*	*B*	*SE*	*CI(LL, UL)*	*B*	*SE*	*CI(LL, UL)*	*B*	*SE*	*CI(LL, UL)*	*B*	*SE*	*CI(LL, UL)*
Age	0.17**	0.06	(0.04, 0.30)	0.02	0.05	(−0.07, 0.12)	−0.01	0.07	(−0.14, 0.12)	0.01	0.07	(−0.13, 0.14)	0.07	0.06	(−0.05, 0.19)
Mentoring	0.46***	0.04	(0.37, 0.54)	0.40***	0.04	(0.31, 0.48)	0.06	0.07	(−0.07, 0.19)	−0.26***	0.07	(−0.39, −0.12)	0.57***	0.06	(0.45, 0.69)
Self-efficacy				0.39***	0.06	(0.28, 0.50)	0.47***	0.09	(0.29, 0.64)	0.89***	0.09	(0.71, 1.07)	0.60***	0.08	(0.43, 0.76)
Gender				0.15*	0.06	(0.03, 0.27)	−0.47***	0.08	(−0.63, −0.30)	−0.61***	0.09	(−0.78, −0.44)	0.25**	0.08	(0.10, 0.40)
Mentoring X Gender				−0.49***	0.08	(−0.66, −0.32)	0.30*	0.13	(0.04, 0.56)	0.42**	0.14	(0.16,0.69)	−0.29*	0.12	(−0.53, −0.05)
Self-efficacy X Gender				0.50***	0.11	(0.28, 0.71)	−0.51***	0.18	(−0.86, −0.16)	−0.85***	0.19	(−1.21, −0.48)	0.35*	0.17	(0.02, 0.68)
Model statistics	*R*^2^ = 0.44, *F*(2, 186) = 72.77***			*R*^2^ = 0.72, *F*(6, 182) = 78.82***			*R*^2^ = 0.31, *F*(6, 182) = 13.40***			*R*^2^ = 0.43, *F*(6, 182) = 22.61***			*R*^2^ = 0.73, *F*(6, 182) = 81.66***		
ΔR^2^ (for Mentoring X Gender)				Δ*R*^2^ = 0.02, *F*(1, 182) = 5.49*			Δ*R*^2^ = 0.02, *F*(1, 182) = 5.49*			Δ*R*^2^ = 0.03, *F*(1, 182) = 9.76*			Δ*R*^2^ = 0.01, *F*(1, 182) = 5.73*		
ΔR^2^ (for Self-efficacy X Gender)				Δ*R*^2^ = 0.03, *F*(1, 182) = 8.11**			Δ*R*^2^ = 0.03, *F*(1, 182) = 8.11**			Δ*R*^2^ = 0.06, *F*(1, 182) = 20.44***			Δ*R*^2^ = 0.01, *F*(1, 182) = 4.35*		

***Conditional effects of mentoring***				***B***	***SE***	***CI(LL, UL)***	***B***	***SE***	***CI(LL, UL)***	***B***	***SE***	***CI(LL, UL)***	***B***	***SE***	***CI(LL, UL)***

Female				0.66***	0.07	(0.53, 0.80)	−0.11	0.09	(−0.29, 0.08)	−0.49***	0.10	(−0.68, −0.29)	0.73***	0.09	(0.56, 0.90)
Male				0.17**	0.05	(0.08, 0.27)	0.20	0.09	(0.02, 0.37)	−0.07	0.10	(−0.25, 0.12)	0.44***	0.08	(0.27, 0.61)

***Conditional effects of self-efficacy***				***B***	***SE***	***CI(LL, UL)***	***B***	***SE***	***CI(LL, UL)***	***B***	***SE***	***CI(LL, UL)***	***B***	***SE***	***CI(LL, UL)***

Female				0.12	0.08	(−0.04, 0.28)	0.75***	0.15	(0.44, 1.05)	1.35***	0.16	(1.03, 1.66)	0.40**	0.14	(0.12, 0.69)
Male				0.62***	0.07	(0.47, 0.76)	0.24*	0.10	(0.05, 0.43)	0.50***	0.10	(0.31, 0.70)	0.76***	0.09	(0.58, 0.93)

***Conditional indirect effects***				***B***	***Boot SE***	***Boot CI (LL, UL)***	***B***	***Boot SE***	***Boot CI (LL, UL)***	***B***	***Boot SE***	***Boot CI (LL, UL)***	***B***	***Boot SE***	***Boot CI (LL, UL)***

Female				0.05	0.03	(−0.01, 0.12)	0.33	0.06	(0.22, 0.45)	0.59	0.08	(0.43, 0.74)	0.18	0.05	(0.08, 0.28)
Male				0.28	0.04	(0.20, 0.36)	0.10	0.05	(0.02, 0.19)	0.22	0.06	(0.11, 0.34)	0.33	0.06	(0.20, 0.45)

***Index of moderated mediation***				***B***	***Boot SE***	***Boot CI (LL, UL)***	***B***	***Boot SE***	***Boot CI (LL, UL)***	***B***	***Boot SE***	***Boot CI (LL, UL)***	***B***	***Boot SE***	***Boot CI (LL, UL)***

Gender				0.23	0.05	(0.12, 0.33)	−0.23	0.07	(−0.36, −0.10)	−0.37	0.09	(−0.54, −0.20)	0.15	0.07	(0.01, 0.29)

* *p* < 0.05, ** *p* < 0.01, *** *p* < 0.001. Gender (Female = 1, Male = 0).

We further proposed in Hypothesis 2 that mentoring predicts intangible entrepreneurial outcomes including (a) satisfaction of need for autonomy, (b) work satisfaction, and the (c) intention to stay in self-employment. The findings revealed significant positive associations of mentoring with satisfaction of need for autonomy (*B* = 0.40, *p* < 001) and intention to stay in self-employment (*B* = 0.57, *p* < 001). Whereas mentoring had insubstantial effects on intrinsic work satisfaction, it was negatively associated with extrinsic work satisfaction (*B* = −0.26, *p* < 0.001). The insignificant association of mentoring and intrinsic work satisfaction suggests that this relationship was fully mediated by self-efficacy; or what is also known as indirect-only mediation (Zhao et al., 2010). On the other hand, self-efficacy had positive effects on all the intangible entrepreneurial outcomes: satisfaction of need for autonomy (*B* = 0.39, *p* < 0.001, intrinsic work satisfaction (*B* = 0.47, *p* < 0.001), extrinsic work satisfaction (*B* = 0.89, *p* < 0.001), and intention to stay in self-employment (*B* = 0.60, *p* < 0.001). In support of Hypothesis 3, our results show that self-efficacy mediated the association between mentoring and the three intangible outcomes as reflected in the indices of the moderated mediation: satisfaction of need for autonomy (*B* = 0.23, *Boot CI* = 0.12, 0.33), intrinsic work satisfaction (*B* = −0.23, *Boot CI* = −0.36, −0.10), extrinsic work satisfaction (*B* = −0.37, *CI* = −0.54, −0.20), and intention to stay in self-employment (*B* = 0.15, *Boot SE* = 0.01, 0.29).

**FIGURE 2:**
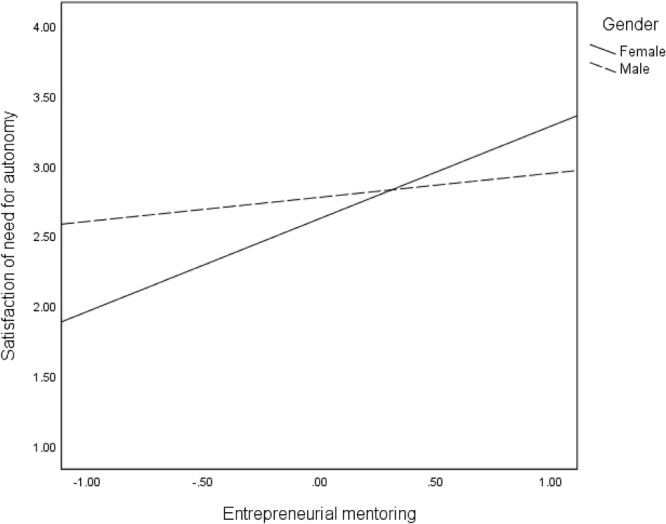
Effects of entrepreneurial mentoring on satisfaction of need for autonomy for females and males.

**FIGURE 3:**
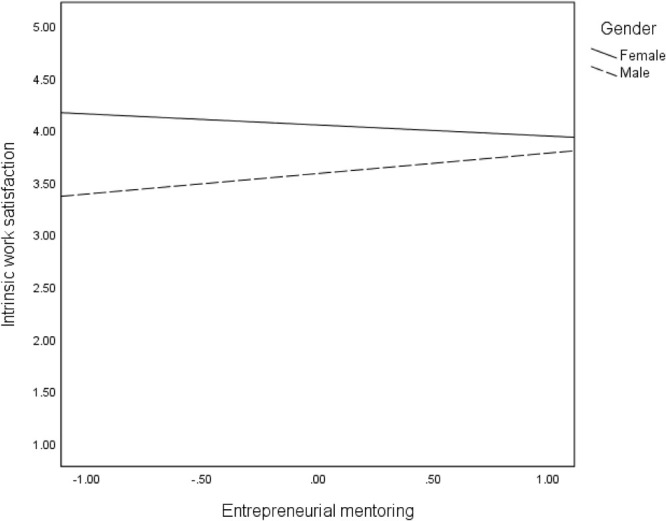
Effects of mentoring on intrinsic work satisfaction for females and males.

**FIGURE 4:**
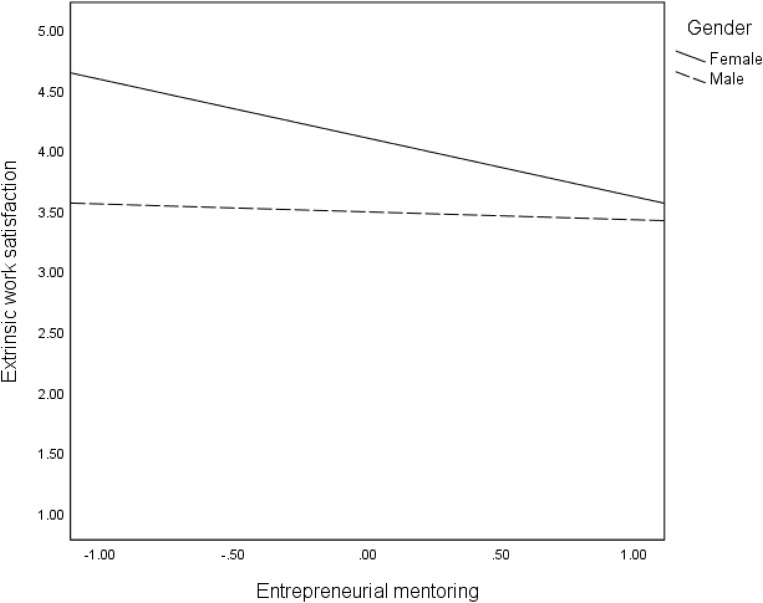
Effects of mentoring on extrinsic work satisfaction for females and males.

**FIGURE 5:**
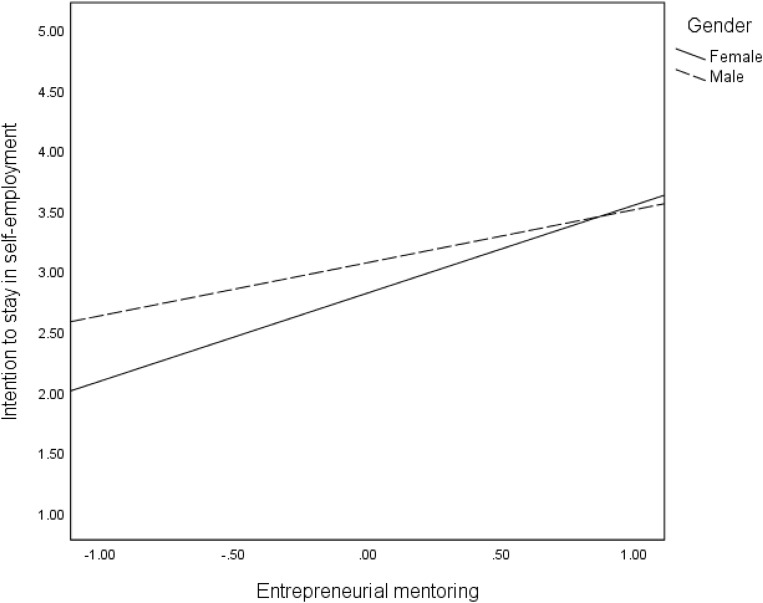
Effects of mentoring on intention to stay in self-employment for females and males.

We proposed that the direct effects (Hypothesis 4) of mentoring on the three intangible outcomes are moderated by gender. The findings in Table 2 revealed significant interaction effects of mentoring and gender on all intangible entrepreneurial outcomes: satisfaction of need for autonomy (*B* = −0.49, *p* < 001), intrinsic work satisfaction (*B* = 0.30, *p* < 0.05), extrinsic work satisfaction (*B* = 0.42, *p* < 0.01), and intention to stay in self-employment (*B* = −29, *p* < 05). These interaction effects are reflected in the regression plots in Figures 2–5. As can be seen in Figures 2, 5 and from the corresponding conditional effects of mentoring in Table 2, satisfaction of the need for autonomy and intention to stay in self-employment were lower for females than males at low levels of mentoring. The trend reversed at high levels of mentoring such that satisfaction of the need for autonomy and intention to stay in self-employment for females were higher than for males. The trend seems to be different when it comes to the intrinsic and extrinsic aspects of work satisfaction. Concerning the intrinsic aspect (Figure 3), females reported higher satisfaction than males, which remained quite the same at all levels of mentoring. However, intrinsic work satisfaction for males tended to move closer to that of females at higher levels of mentoring. Whereas females report generally high levels of extrinsic satisfaction that males, the satisfaction tends to lower at high levels if mentoring while that of males remains unchanged at all levels of mentoring (Figure 4).

**FIGURE 6:**
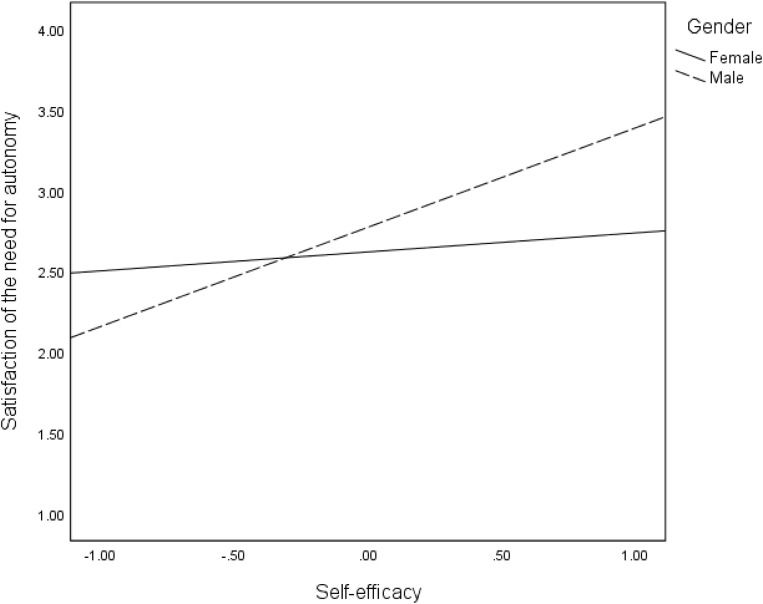
Effects of self-efficacy on satisfaction of need for autonomy for females and males.

**FIGURE 7:**
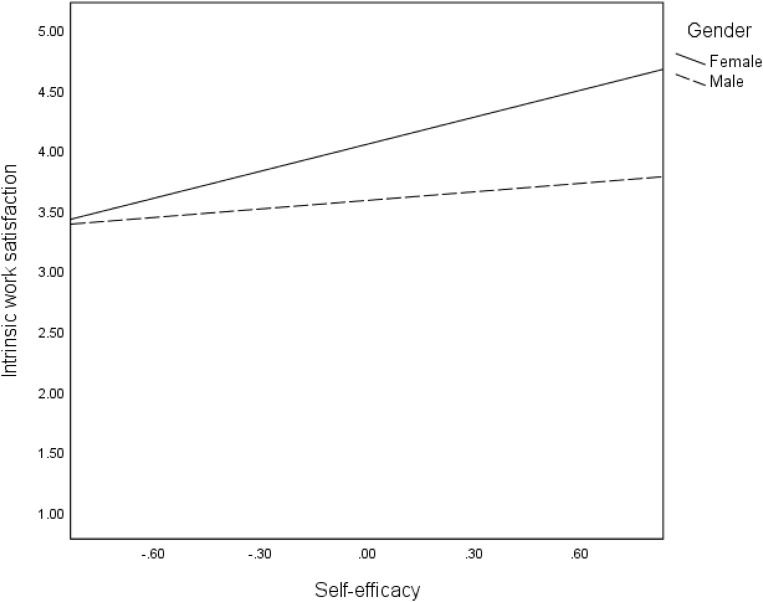
Effects of self-efficacy on intrinsic work satisfaction for females and males.

**FIGURE 8:**
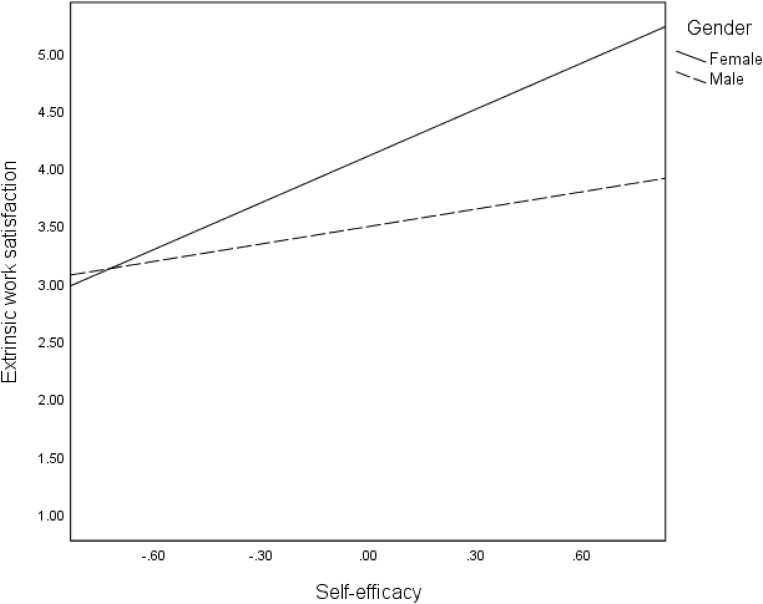
Effects of self-efficacy on extrinsic work satisfaction for females and males.

**FIGURE 9:**
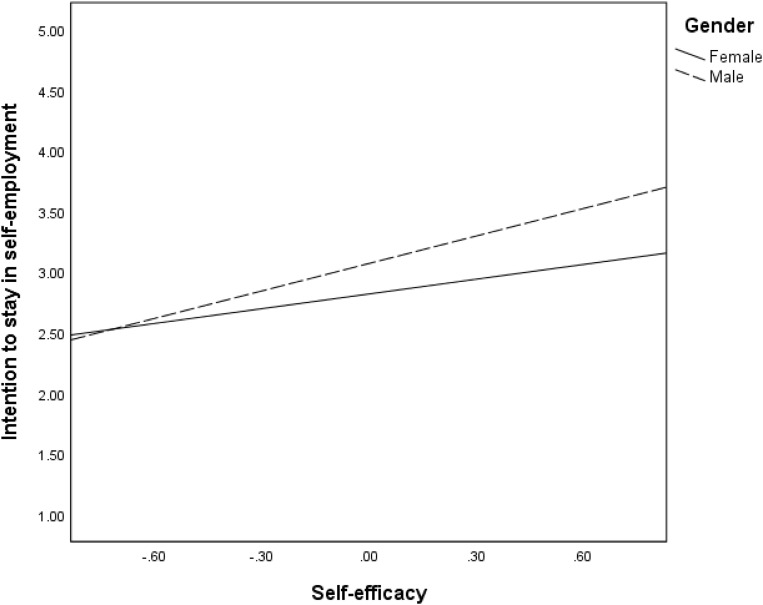
Effects of self-efficacy on intention to stay in self-employment for females and males.

In hypothesis 5, we proposed that indirect effects of mentoring on intangible entrepreneurial outcomes are moderated by gender. The interactive effects of self-efficacy and gender were significant for all the intangible entrepreneurial outcomes: satisfaction of the need for autonomy (*B* = 0.50, *p* < 001), intrinsic work satisfaction (*B* = −0.51, *p* < 001), extrinsic work satisfaction (*B* = −0.85, *p* < 0.001) and intention to stay in self-employment (*B* = 0.35, *p* < 05). The moderations are confirmed in the regression plots in Figures 6–9, as well as the conditional effects of self-efficacy in Table 2. Plots in Figures 6, 9 indicate that that males had substantially higher satisfaction of the need for autonomy and intention to stay in self-employment, respectively, than the females at high levels of self-efficacy. On the contrary, plots in Figures 7, 8 show that females reported a higher level of intrinsic and extrinsic work satisfaction than the males at high levels of self-efficacy.

The conditional indirect effects and indices of moderated mediation in Table 2 confirmed Hypothesis 5. The indirect effects of mentoring through self-efficacy were moderated by gender for all three intangible entrepreneurial outcomes. The indirect effects on the satisfaction of the need for autonomy were significant for males (*B* = 0.28, *Boot CI* = 0.20, 0.36) and not for females. On the other hand, the indirect effects on intrinsic work satisfaction were significant for both females (*B* = 0.33, *Boot CI* = 0.22, 0.45) males (*B* = 0.10, *Boot CI* = 0.02, 0.19), although stronger for females. A similar trend is observed for extrinsic work satisfaction. Finally, the indirect effects on intention to stay in self-employment were significant for both males (*B* = 0.33, *Boot CI* = 0.20, 0.45) and females (*B* = 0.18, *Boot CI* = 0.08, 0.28), but stronger for males.

## Discussion

The present study highlights the role of entrepreneurial mentoring and self-efficacy in the attainment of intangible entrepreneurial outcomes. We argue that besides the development of entrepreneurial skills that lead to objective success, mentoring nascent entrepreneurs is directly and indirectly associated with their level of satisfaction of the need for autonomy, their work satisfaction (both intrinsic and extrinsic), and consequently the desire to stay in self-employment. Individuals seek different goals by engaging in entrepreneurial activities; hence success indicators are not uniform among entrepreneurs. The subjective aspects of success could be as important as the objective aspects given that they present what the entrepreneurs themselves value (Dej and Gorgievski, 2012; Wach et al., 2016). Moreover, because today’s careers tend to be value driven (Hall, 2002), intangible and more especially intrinsic outcomes become enormously important (Otto et al., 2017). Therefore, entrepreneurial support efforts including mentoring should be directed toward attainment of not only the objective but also the subjective outcomes.

Entrepreneurial mentoring plays an important role in enterprise growth and success through problem identification, providing solutions, information and emotional support, persuasion, and many other functions (Cull, 2006; St-Jean, 2012; Radu Lefebvre and Redien-Collot, 2013). Our results suggest that these different forms of support help in the development of entrepreneurs’ general self-efficacy as well as the achievement of some of the intangible entrepreneurial outcomes. Through practical learning and guidance from experienced entrepreneurs, the novice entrepreneurs acquire the ability to make independent decisions, become more creative, innovative, more alert to opportunities. These consequently support the realization of different goals including the need for autonomy and work satisfaction. Yet attainment of such intrinsic goals, in line with self-determination theory (Deci et al., 2001), can stimulate the desire to stay in self-employment.

The findings of the present study further extend our theoretical understanding of how mentoring results in entrepreneurial success and persistence. First, our results build on the entrepreneurial socialization approaches (Starr and Fondas, 1992; St-Jean, 2012; Man, 2019) which highlight that entrepreneurial competencies are developed through education and training. Our study further builds on the Social Cognitive Career Theory (Hackett and Lent, 1992; Lent and Brown, 2013) and self-efficacy theory (Stajkovic and Luthans, 1998; Bandura, 2010) which emphasize that self-efficacy is one of the competencies that are strengthened by learning and yet is an important predictor of career outcomes including satisfaction and persistence. Supporting these assumptions, our findings demonstrated that self-efficacy is an important underlying mechanism through which mentoring fosters entrepreneurial outcomes. In the field of entrepreneurship, however, it has been suggested that the learning goal orientation of the protégé as well as the match in characteristics of the mentor and mentee are important for improving entrepreneurial self-efficacy of a novice entrepreneur (St-Jean et al., 2018). In general, however, whereas mentoring improves entrepreneurs’ skills to perform entrepreneurial tasks including business planning, identifying opportunities, resources mobilization and management, such empowerment does not necessarily imply that individuals will engage in these tasks with the desired level of effort. But it helps when entrepreneurial mentoring alongside developing these competencies also enhances psychological resources, specifically self-efficacy, which then becomes a driver for the application of the skills acquired and persistence in entrepreneurial actions.

Our findings further contribute to the literature on the role of gender in entrepreneurship and entrepreneurial socialization. Extant literature shows that there are gender differences in involvement and persistence in entrepreneurial activities (Scott and Shu, 2017; Smith and Tolbert, 2018; Guzman and Kacperczyk, 2019; Oppedal and Garcia, 2019). In the present study, we sought to contribute to this domain in the entrepreneurial literature by establishing whether men and women benefit equally from entrepreneurial mentoring and self-efficacy in terms of achieving intangible outcomes of entrepreneurship. Our findings reveal three but interrelated issues. First, men reported higher satisfaction of the need for autonomy and intention to stay in self-employment as well as higher level of self-efficacy, while women reported higher levels of both intrinsic and extrinsic work satisfaction. This implies that men tend to achieve a higher level of independence in work when engaged in an entrepreneurial activity. This independence may stimulate the commitment to self-employment in line with the assumptions of the self-determination theory (Deci et al., 2001; García Calvo et al., 2010; Baluku et al., 2018b). This may not apply equally to women, especially in a cultural context that predominantly embraces collectivism and patriarchy. Hence, even when engaged in entrepreneurship, women may still be required to depend on their husbands or parents when it comes to making key decisions for the business. In this direction, previous research in this context has observed that for example husbands play an important role in women’s entrepreneurial activity and performance (Wolf and Frese, 2018). Consequently, although women can be satisfied with their work, they may not necessarily achieve autonomy which might eventually also lower their intentions to stay in self-employment.

Second, and contrary to the above finding, the moderation effects of gender on the association between mentoring and intangible entrepreneurial outcomes reveal that the effect of mentoring on the satisfaction of the need for autonomy and intention to stay in self-employment is stronger for women. Hence, mentoring has the potential to enabling women entrepreneurs to gain independence in their work as well as increasing their likelihoods to persisting in entrepreneurial activities for a longer time. Nonetheless, when considering self-efficacy, men still reported a higher level of satisfaction of the need for autonomy and intention to stay in self-employment. A similar pattern of findings is observed regarding the moderation effects of gender on the indirect effects of mentoring through self-efficacy. Although the changes in *R*^2^ are quite low for most of our interaction effects, our findings suggest that men and women benefit from entrepreneurial mentoring differently. In relation to both intrinsic and extrinsic work satisfaction, women benefit more from mentoring if the mentoring process improves their self-efficacy. This is consistent with earlier findings suggesting that women could benefit more than men in entrepreneurial terms from self-efficacy (Kickul et al., 2008). Whereas men also benefit this way, the effects are stronger for women. Regression plots in Figure 8 particularly show that improvement in self-efficacy in female could sharply improve their extrinsic work satisfaction. The mechanism of how this occurs needs to be explored further. However, in relation to satisfaction of the need for autonomy and intention to stay in self-employment, women benefit more directly from mentoring, while the benefits for men are higher if mentoring strengthens their self-efficacy. The dynamics causing these differences need to be explored, especially taking into consideration of the social and cultural context. Our results support previous research that has found moderated mediation effects of mentoring on entrepreneurial outcome variables such as entrepreneurial intention, via self-efficacy and moderated by gender (BarNir et al., 2011).

One possible explanation for the weak mediation effects of self-efficacy in the association between mentoring for women and particularly the satisfaction of the need for autonomy could be the overall low number of female entrepreneurial role models and mentors as well as the cultural aspect of patriarchy that may deny women the desired level of autonomy even when they have access to good entrepreneurship mentoring. However, having female role models and mentors does not necessary imply that females will benefit much more than when they have male role models and mentors (Goh et al., 2007). Future research should, therefore, investigate the success of entrepreneurial mentoring for women and men in different cultural contexts as well as how these cultural contexts influence different facets of entrepreneurial success among men and women entrepreneurs. In practical terms, entrepreneurial training and mentoring interventions, at least in the Ugandan context, need to focus on strengthening entrepreneurial self-efficacy of the protégés. Moreover, there is a need to design specific interventions – potentially even conducted by female role models – addressing the self-efficacy and autonomy issues among women entrepreneurs.

### Limitations

Despite the theoretical and practical contributions discussed above, our study is not without limitations. First, we used cross-sectional data to test our hypotheses. Caused by the fact that mentoring, self-efficacy and the three intangible outcomes of entrepreneurship were measured at the same time, we cannot firmly claim that the intangible outcomes accrue from mentoring and self-efficacy. Moreover, our sample may not be representative of young entrepreneurs in less developed countries given that the study was conducted in one major city in Uganda. It should also be noted that changes in *R*^2^ are quite small for most of the moderation effects. However, overall, our results are in line with previous studies that associated entrepreneurship education and training to the development of entrepreneurial self-efficacy and entrepreneurial outcomes (Nabi and Liñán, 2011; Nabi et al., 2017; Baluku et al., 2019b; Hahn et al., 2019). We recommend that future studies on the effectiveness of entrepreneurial mentoring and other learning approaches could benefit from longitudinal, cross-country, and probably multi-year studies given that entrepreneurial ecosystems that affect the effectiveness of mentoring vary across countries/cities and the amount of time required for protégés applying the acquired knowledge and skills and eventual attainment of entrepreneurial success. In addition, we measured informal mentoring. However, the possibility that some participants also had access to formal mentoring cannot be ruled out, yet we did not control for the effects of formal mentoring. Future studies could benefit from an effort to segregate the effects of the two forms of entrepreneurial mentoring.

Another potential limitation of the study relates to the use of self-report measures. This is in addition to our focus on only the subjective intangible outcomes of entrepreneurship. Subjective outcomes of entrepreneurial activities, specifically satisfaction, are linked to or affected by the performance level of the business (Cooper and Artz, 1995; Hmieleski and Corbett, 2008; Carree and Verheul, 2012). The shortcoming of the present study is that we did not control for the effect of firm performance on subjective outcomes. This also suggests that some of the subjective outcomes can also accrue not directly from entrepreneurship mentoring but indirectly through the impact of mentoring on facets of objective entrepreneurial success. It could be interesting for future research to use both objective and subjective measures as well as examining the possible mediation and moderating effects of objective success on the association between mentoring and subjective success indicators.

## Conclusion

The present research has demonstrated the differential impact of mentoring on intangible outcomes of entrepreneurship among men and women. The study has also validated self-efficacy as an underlying mechanism for the realization of the impact of entrepreneurial mentoring. Consequently, this study contributes to the understanding of the effectiveness of entrepreneurship education and learning interventions among women and men in the context of a less developed country as well as in the cultural context of collectivism and patriarchy. In doing so, we were able to discover the gaps in the effectiveness of entrepreneurship mentoring and learning and make a call for interventions that strengthen the entrepreneurial self-efficacy and autonomy of novice entrepreneurs but particularly of novice women entrepreneurs which can be achieved by using successful women entrepreneurs and mentors.

## Data Availability Statement

The datasets generated for this study are available on request to the corresponding author.

## Ethics Statement

Ethical review and approval was not required for the study on human participants in accordance with the local legislation and institutional requirements. The patients/participants provided their written informed consent to participate in this study.

## Author Contributions

MB is the main author and was in charge of developing the manuscript and data collection. LM participated in the data collection and analysis as well as editing of the manuscript. KO participated in conceptualizing the data, data analysis, and editing of the manuscript. All authors contributed to the article and approved the submitted version.

## Conflict of Interest

The authors declare that the research was conducted in the absence of any commercial or financial relationships that could be construed as a potential conflict of interest.

## References

[B1] Ahmed T. Chandran V. G. R. Klobas J. (2017). Specialized entrepreneurship education: does it really matter? Fresh evidence from Pakistan. *Int. J. Entrep. Behav. Res.* 23 4–19. 10.1108/IJEBR-01-2016-0005

[B2] Ahsan M. Zheng C. DeNoble A. Musteen M. (2018). From student to entrepreneur: how mentorships and affect influence student venture launch. *J. Small Bus. Manag.* 56 76–102. 10.1111/jsbm.12362

[B3] Ajzen I. (2002). Perceived behavioral control, self-efficacy, locus of control, and the theory of planned behavior. *J. Appl. Soc. Psychol.* 80 2918–2940. 10.1111/j.1559-1816.2002.tb00236.x

[B4] Apitzsch U. (2003). Gaining autonomy in self-employment processes: The biographical embeddedness of women’s and migrants’ business. *Int. Rev. Sociol.* 13 163–182. 10.1080/0390670032000087041

[B5] Artinger S. Powell T. C. (2016). Entrepreneurial failure: Statistical and psychological explanations. *Strateg. Manag. J.* 37 1047–1064. 10.1002/smj.2378

[B6] Autio E. Keeley H. Klofsten M. R. Parker G. C. Hay M. (2001). Entrepreneurial intent among students in scandinavia and in the USA. *Enter. Innov. Manag. Stud.* 2 145–160. 10.1080/14632440110094632

[B7] Avey J. B. Luthans F. Smith R. M. Palmer N. F. (2010). Impact of positive psychological capital on employee well-being over time. *J. Occup. Health Psychol.* 15 17–28. 10.1037/a0016998 20063956

[B8] Baluku M. M. Kikooma J. F. Bantu E. Otto K. (2018a). Psychological capital and entrepreneurial outcomes: the moderating role of social competences of owners of micro-enterprises in East Africa. *J. Glob. Entrep. Res.* 8:26 10.1186/s40497-018-0113-7

[B9] Baluku M. M. Kikooma J. F. Otto K. (2018b). Positive mindset and entrepreneurial outcomes: the magical contributions of psychological resources and autonomy. *J. Small Bus. Entrep.* 30 473–498. 10.1080/08276331.2018.1459017

[B10] Baluku M. M. Leonsio M. Bantu E. Otto K. (2019a). The impact of autonomy on the relationship between mentoring and entrepreneurial intentions among youth in Germany, Kenya, and Uganda. *Int. J. Entrep. Behav. Res.* 25 170–192. 10.1108/IJEBR-10-2017-0373

[B11] Baluku M. M. Matagi L. Musanje K. Kikooma J. F. Otto K. (2019b). Entrepreneurial socialization and psychological capital: Cross-cultural and multigroup analyses of impact of mentoring, optimism, and self-efficacy on entrepreneurial intentions. *Entrep. Educ. Pedagog.* 2 5–42. 10.1177/2515127418818054

[B12] Bandura A. (1997). *Self-Efficacy: The Exercise of Control.* New York, NY: Worth Publishers, 10.5860/CHOICE.35-1826

[B13] Bandura A. (2010). “Self-Efficacy,” in *The Corsini Encyclopedia of Psychology*, eds Weiner B. I. Craighead W. E. (Hoboken, NJ: John Wiley & Sons, Inc), 1–3. 10.1002/9780470479216.corpsy0836

[B14] BarNir A. Watson W. E. Hutchins H. M. (2011). Mediation and moderated mediation in the relationship among role models, self-efficacy, entrepreneurial career intention, and gender. *J. Appl. Soc. Psychol.* 41 270–297. 10.1111/j.1559-1816.2010.00713.x

[B15] Baron R. A. Franklin R. J. Hmieleski K. M. (2016a). Why entrepreneurs often experience low, not high, levels of stress: The joint effects of selection and psychological capital. *J. Manage.* 42 742–768. 10.1177/0149206313495411

[B16] Baron R. A. Mueller B. A. Wolfe M. T. (2016b). Self-efficacy and entrepreneurs’ adoption of unattainable goals: The restraining effects of self-control. *J. Bus. Ventur.* 31 55–71. 10.1016/j.jbusvent.2015.08.002

[B17] Baron R. A. Henry R. A. (2011). “Entrepreneurship: the genesis of organizations,” in *APA Handbook of Industrial and Organizational Psychology, Vol 1: Building and Developing the Organization*, ed. Zedeck S. (Washington, DC: American Psychological Association), 241–273. 10.1037/12169-008

[B18] Beckett B. J. (2010). Mentorship is key to career success. *Strateg. Financ.* 92 21–122.

[B19] Berglund V. Johansson Sevä I. Strandh M. (2015). Subjective well-being and job satisfaction among self-employed and regular employees: does personality matter differently? *J. Small Bus. Entrep.* 28 1–19. 10.1080/08276331.2015.1115699

[B20] Bergman N. Rosenblatt Z. Erez M. De-Haan U. (2011). Gender and the effects of an entrepreneurship training programme on entrepreneurial self-efficacy and entrepreneurial knowledge gain. *Int. J. Entr. Small Bus.* 13 38–54. 10.1504/IJESB.2011.040415

[B21] Binder M. Coad A. (2013). Life satisfaction and self-employment: a matching approach. *Small Bus. Econ.* 40 1009–1033. 10.1007/s11187-011-9413-9

[B22] Blau G. J. (1985). The measurement and prediction of career commitment. *J. Occup. Psychol*. 58 277–288. 10.1111/j.2044-8325.1985.tb00201.x

[B23] Blau G. J. (1988). Further exploring the meaning and measurement of career commitment. *J. Vocat. Behav*. 32 284–297. 10.1016/0001-8791(88)90020-6

[B24] Boyd N. G. Vozikis G. S. (1994). The influence of self-efficacy on the development of entrepreneurial intentions and actions. *Entrep. Theory Pract.* 18 63–77. 10.1080/02640410152475847 11522147

[B25] Brodie J. Van Saane S. H. Osowska R. (2017). Help wanted: Exploring the value of entrepreneurial mentoring at start-up. *Ind. High. Educ.* 31 122–131. 10.1177/0950422217691666

[B26] Bruni A. Gheradi S. Poggio B. (2005). *Gender and Entrepreneurship: An Ethnographic Approach–Attila Bruni, Silvia Gherardi, Barbara Poggio–Google Books.* London: Routledge.

[B27] Bullough A. Renko M. Myatt T. (2014). Danger zone entrepreneurs: The importance of resilience and self-efficacy for entrepreneurial intentions. *Entrep. Theory Pract.* 38 473–499. 10.1111/etap.12006

[B28] Cardon M. S. Kirk C. P. (2015). Entrepreneurial passion as mediator of the self-efficacy to persistence relationship. *Entrep. Theory Pract.* 39 1027–1050. 10.1111/etap.12089

[B29] Carree M. A. Verheul I. (2012). What makes entrepreneurs happy? Determinants of satisfaction among founders. *J. Happiness Stud.* 13 371–387. 10.1007/s10902-011-9269-3

[B30] Cascio T. Gasker J. (2001). Everyone has a shining side: Computer-mediated mentoring in social work education. *J. Soc. Work Educ.* 37 283–293. 10.1080/10437797.2001.10779054

[B31] Chang S. J. Van Witteloostuijn A. Eden L. (2010). From the editors: common method variance in international business research. *J. Int. Bus. Stud.* 41 178–184. 10.1057/jibs.2009.88

[B32] Chao G. T. Walz P. Gardner P. D. (1992). Formal and informal mentorships: a comparison on mentoring functions and contrast with nonmentored counterparts. *Pers. Psychol.* 45 619–636. 10.1111/j.1744-6570.1992.tb00863.x

[B33] Cooper A. C. Artz K. W. (1995). Determinants of satisfaction for entrepreneurs. *J. Bus. Ventur.* 10 439–457. 10.1016/0883-9026(95)00083-K

[B34] Cope J. (2011). Entrepreneurial learning from failure: An interpretative phenomenological analysis. *J. Bus. Ventur.* 26 604–623. 10.1016/j.jbusvent.2010.06.002

[B35] Croson D. C. Minniti M. (2012). Slipping the surly bonds: The value of autonomy in self-employment. *J. Econ. Psychol.* 33 355–365. 10.1016/j.joep.2011.05.001

[B36] Culbertson S. S. Smith M. R. Leiva P. I. (2011). Enhancing entrepreneurship: The role of goal orientation and self-efficacy. *J. Career Assess.* 19 115–129. 10.1177/1069072710385543

[B37] Cull J. (2006). Mentoring young entrepreneurs: What leads to success? *Int. J. Evid. Based Coach. Mentor.* 4 8–18.

[B38] Datta P. B. Gailey R. (2012). Empowering Women through social entrepreneurship: Case study of a women’s cooperative in India. *Entrep. Theory Pract.* 36 569–587. 10.1111/j.1540-6520.2012.00505.x

[B39] De Jager W. Kelliher C. Peters P. Blomme R. Sakamoto Y. (2016). Fit for self-employment? An extended Person-Environment Fit approach to understand the work-life interface of self-employed workers. *J. Manag. Organ.* 22 797–816. 10.1017/jmo.2016.41

[B40] De Neve J. Ward G. W. (2017). *Happiness at work. Saïd Business School WP 2017-07.* Available online at: https://ssrn.com/abstract=2943318 (accessed February 1, 2017).

[B41] Deale C. S. Lee S. H. Bae J. I. (2020). Making mentoring meaningful: hospitality and tourism students’ perceptions of mentoring. *J. Teach. Travel Tour.* 20 1–22. 10.1080/15313220.2019.1601051

[B42] Deci E. L. Ryan R. M. (2000). The “what” and “why” of goal pursuits: human needs and the self-determination of behavior. *Psychol. Inq.* 11 227–268. 10.1207/S15327965PLI1104_01

[B43] Deci E. L. Ryan R. M. (2008). Self-determination theory: a macrotheory of human motivation, development, and health. *Can. Psychol. Can.* 49 182–185. 10.1037/a0012801

[B44] Deci E. L. Ryan R. M. Gagné M. Leone D. R. Usunov J. Kornazheva B. P. (2001). Need satisfaction, motivation, and well-being in the work organizations of a former eastern bloc country: a cross-cultural study of self-determination. *Personal. Soc. Psychol. Bull.* 27 930–942. 10.1177/0146167201278002

[B45] Dej D. Gorgievski M. (2012). Subjective entrepreneurial success – Development of a multi dimensional measurement instrument. *Acad. Manag. Proc.* 2012:18244 10.5465/ambpp.2012.18244abstract

[B46] DeTienne D. R. Shepherd D. A. De Castro J. O. (2008). The fallacy of “only the strong survive”: The effects of extrinsic motivation on the persistence decisions for under-performing firms. *J. Bus. Ventur.* 23 528–546. 10.1016/j.jbusvent.2007.09.004

[B47] Dijkhuizen J. Veldhoven M. van Schalk R. (2016). Four types of well-being among entrepreneurs and their relationships with business performance. *J. Entrep.* 25 184–210. 10.1177/0971355716650369

[B48] Dimov D. (2010). Nascent entrepreneurs and venture emergence: Opportunity confidence, human capital, and early planning. *J. Manag. Stud.* 47 1123–1153. 10.1111/j.1467-6486.2009.00874.x

[B49] Duggleby W. Cooper D. Penz K. (2009). Hope, self-efficacy, spiritual well-being and job satisfaction. *J. Adv. Nurs.* 65 2376–2385. 10.1111/j.1365-2648.2009.05094.x 19737323

[B50] Edelman L. F. Brush C. G. Manolova T. S. Greene P. G. (2010). Start-up motivations and growth intentions of minority nascent entrepreneurs. *J. Small Bus. Manag.* 48 174–196. 10.1111/j.1540-627X.2010.00291.x

[B51] Edwards L. J. Muir E. J. (2005). Promoting entrepreneurship at the University of Glamorgan through formal and informal learning. *J. Small Bus. Enterp. Dev.* 12 613–626. 10.1108/14626000510628261

[B52] Ensher E. A. Murphy S. E. Vance C. M. (2000). Mentoring and self-management career strategies for entrepreneurs. *Int. J. Entrep. Innov.* 1 99–108. 10.5367/000000000101298595

[B53] Fayolle A. Gailly B. (2015). The impact of entrepreneurship education on entrepreneurial attitudes and intention: Hysteresis and persistence. *J. Small Bus. Manag.* 53 75–93. 10.1111/jsbm.12065

[B54] Felfe J. Schmook R. Schyns B. Six B. (2008). Does the form of employment make a difference?-Commitment of traditional, temporary, and self-employed workers. *J. Vocat. Behav.* 72 81–94. 10.1016/j.jvb.2007.10.008

[B55] Fisher C. D. (2010). Happiness at Work. *Int. J. Manag. Rev.* 12 384–412. 10.1111/j.1468-2370.2009.00270.x

[B56] Forbes D. P. (2005). The effects of strategic decision making on entrepreneurial self-efficacy. *Entrep. Theory Pract.* 29 599–626. 10.1111/j.1540-6520.2005.00100.x

[B57] García Calvo T. Cervelló E. Jiménez R. Iglesias D. Moreno Murcia J. A. (2010). Using self-determination theory to explain sport persistence and dropout in adolescent athletes. *Span. J. Psychol.* 13 677–684. 10.1017/S1138741600002341 20977017

[B58] Gimmon E. (2014). Mentoring as a practical training in higher education of entrepreneurship. *Educ. Train.* 56 814–825. 10.1108/ET-02-2014-0006

[B59] Goel S. Karri R. (2006). Entrepreneurs, effectual logic, and over-trust. *Entrep. Theory Pract.* 30 477–493. 10.1111/j.1540-6520.2006.00131.x

[B60] Goh D. Ogan C. Ahuja M. Herring S. C. Robinson J. C. (2007). Being the same isn’t enough: impact of male and female mentors on computer self-efficacy of college students in it-related fields. *J. Educ. Comput. Res.* 37 19–40. 10.2190/3705-4405-1G74-24T1 22612255

[B61] Gong R. Chen S.-Y. Lee S.-L. (2011). Does mentoring work? The mediating effect of mentoring in China. *Soc. Behav. Pers.* 39 807–824. 10.2224/sbp.2011.39.6.807

[B62] Guzman J. Kacperczyk A. (2019). Gender gap in entrepreneurship. *Res. Policy* 48 1666–1680. 10.1016/j.respol.2019.03.012

[B63] Hackett G. Lent R. W. (1992). Theoretical advances and current inquiry in career psychology. *Handb. Couns. Psychol.* 419–451.

[B64] Hahn D. Minola T. Bosio G. Cassia L. (2019). The impact of entrepreneurship education on university students’ entrepreneurial skills: a family embeddedness perspective. *Small Bus. Econ.* 19 1–29. 10.1007/s11187-019-00143-y

[B65] Hair J. F. Ringle C. M. Sarstedt M. (2011). PLS-SEM: Indeed a Silver Bullet. *J. Mark. Theory Pract.* 19 139–152. 10.2753/MTP1069-6679190202

[B66] Hall D. T. (2002). *Careers In and Out of Organisations.* Thousand Oaks, CA: SAGE Publications, Inc.

[B67] Hasan S. M. Khan E. A. Nabi M. N. U. (2017). Entrepreneurial education at university level and entrepreneurship development. *Educ. Train.* 59 888–906. 10.1108/ET-01-2016-0020

[B68] Hauber F. A. Bruininks R. H. (1986). Intrinsic and extrinsic job satisfaction among direct-care staff in residential facilities for mentally retarded people. *Educ. Psychol. Meas.* 46 95–105. 10.1177/0013164486461009

[B69] Hayes’ A. F. (2013). *Introduction to Mediation, Moderation, and Conditional Process Analysis.* New York, NY: Guilford Press, doi: 978-1-60918-230-4

[B70] Hirschfeld R. R. (2000). Does revising the intrinsic and extrinsic subscales of the minnesota satisfaction questionnaire short form make a difference? *Educ. Psychol. Meas.* 60 255–270. 10.1177/00131640021970493

[B71] Hmieleski K. M. Corbett A. C. (2008). The contrasting interaction effects of improvisational behavior with entrepreneurial self-efficacy on new venture performance and entrepreneur work satisfaction. *J. Bus. Ventur.* 23 482–496. 10.1016/j.jbusvent.2007.04.002

[B72] Ho M.-H. R. Uy M. A. Kang B. N. Y. Chan K.-Y. (2018). Impact of entrepreneurship training on entrepreneurial efficacy and alertness among adolescent youth. *Front. Educ.* 3:13 10.3389/feduc.2018.00013

[B73] Hundley G. (2001). Why and when are the self-employed more satisfied with their work? *Ind. Relat. (Berkeley).* 40 293–316. 10.1111/0019-8676.00209

[B74] Huq A. Gilbert D. (2017). All the world’s a stage: transforming entrepreneurship education through design thinking. *Educ. Train.* 59 155–170. 10.1108/ET-12-2015-0111

[B75] Jain R. Ali S. W. (2013). Self-efficacy beliefs, marketing orientation and attitude orientation of indian entrepreneurs. *J. Entrep.* 22 71–95. 10.1177/0971355712469155

[B76] Judge T. A. Thoresen C. J. Bono J. E. Patton G. K. (2001). The job satisfaction-job performance relationship: A qualitative and quantitative review. *Psychol. Bull.* 127 376–407. 10.1037/0033-2909.127.3.376 11393302

[B77] Karademas E. C. (2006). Self-efficacy, social support and well-being: The mediating role of optimism. *Pers. Individ. Dif.* 40 1281–1290. 10.1016/j.paid.2005.10.019

[B78] Karlsson T. Moberg K. (2013). Improving perceived entrepreneurial abilities through education: Exploratory testing of an entrepreneurial self efficacy scale in a pre-post setting. *Int. J. Manag. Educ.* 11 1–11. 10.1016/j.ijme.2012.10.001

[B79] Kickul J. Wilson F. Marlino D. Barbosa S. D. (2008). Are misalignments of perceptions and self-efficacy causing gender gaps in entrepreneurial intentions among our nation’s teens? *J. Small Bus. Enterp. Dev.* 15 321–335. 10.1108/14626000810871709

[B80] Kolvereid L. (1996). Organisational employment versus self employment: Reasons for career choice intentions. *Entrep. Theory Pract.* 20 23–31. 10.6018/analesps.31.1.161461

[B81] Krueger N. Dickson P. (1994). How believing in ourselves increases risk taking: perceived self-efficacy and opportunity recognition. *Decis. Sci.* 25 385–400. 10.1111/j.1540-5915.1994.tb00810.x

[B82] Krueger N. F. (2007). What lies beneath? The experiential essence of entrepreneurial thinking. *Entrep. Theory Pract.* 31 123–138. 10.1111/j.1540-6520.2007.00166.x

[B83] Kuratko D. F. (2007). Entrepreneurial leadership in the 21st century. *J. Leadersh. Organ. Stud.* 13 1–11. 10.1177/10717919070130040201

[B84] Kyrgidou L. P. Petridou E. (2013). Developing women entrepreneurs&apos; knowledge, skills and attitudes through e-mentoring support. *J. Small Bus. Enterp. Dev.* 20 548–566. 10.1108/JSBED-04-2013-0061

[B85] Langer J. Feeney M. K. Lee S. E. (2019). Employee fit and job satisfaction in bureaucratic and entrepreneurial work environments. *Rev. Public Pers. Adm.* 39 135–155. 10.1177/0734371X17693056

[B86] Larson M. Luthans F. (2006). Potential added value of psychological capital in predicting work attitudes. *J. Leadersh. Organ. Stud.* 13 75–92. 10.1177/10717919070130020601

[B87] Lent R. W. Brown S. D. (2008). Social cognitive career theory and subjective well-being in the context of work. *J. Career Assess.* 16 6–21. 10.1177/1069072707305769

[B88] Lent R. W. Brown S. D. (2013). Social cognitive model of career self-management: toward a unifying view of adaptive career behavior across the life span. *J. Couns. Psychol.* 60 557–568. 10.1037/a0033446 23815631

[B89] Lent R. W. Brown S. D. Hackett G. (1994). Toward a unifying social cognitive theory of career and academic interest, choice, and performance. *J. Vocat. Behav.* 45 79–122. 10.1006/jvbe.1994.1027

[B90] Lent R. W. Brown S. D. Hackett G. (2002). Social cognitive career theory. *Career choice Dev. Appl. Contemp. Theor. to Pract.* 20 255–369. 10.2307/184400

[B91] Lo M. C. Ramayah T. (2011). Mentoring and job satisfaction in Malaysian SMEs. *J. Manag. Dev.* 30 427–440. 10.1108/02621711111126891

[B92] Locke E. A. (1969). What is job satisfaction? *Organ. Behav. Hum. Perform.* 4 309–336. 10.1016/0030-5073(69)90013-0

[B93] Luthans F. (2002). Positive organizational behavior: developing and managing psychological strengths. *Acad. Manag. Exec.* 16 57–72. 10.5465/AME.2002.6640181

[B94] Luthans F. Avolio B. J. Avey J. B. (2007a). *Psychological Capital (PsyCap) Questionnaire (PCQ).* Menlo Park, CA: Mind Garden, Inc.

[B95] Luthans F. Avolio B. J. Avey J. B. Norman S. M. (2007b). Positive psychological capital: measurement and relationship with performance and satisfaction. *Pers. Psychol.* 60 541–572. 10.1111/j.1744-6570.2007.00083.x

[B96] Luthans F. Youssef C. M. Avolio B. J. (2007c). “Psychological capital: Investing and developing positive organizational behavior,” in *Positive Organizational Behavior*, eds Nelson D. Cooper C. L. (London: Sage publications), 9–24. 10.4135/9781446212752.n2

[B97] Luthans F. Luthans K. W. Luthans B. C. (2004). Positive psychological capital: Beyond human and social capital. *Bus. Horiz.* 47 45–50. 10.1016/j.bushor.2003.11.007

[B98] Luthans F. Peterson S. J. (2002). Employee engagement and manager self-efficacy. *J. Manag. Dev.* 21 376–387. 10.1108/02621710210426864

[B99] Luthans F. Youssef C. M. Avolio B. J. (2015). *Psychological Capital and Beyond.* New York, NY: Oxford University Press, 10.1002/smi.2623

[B100] Luthans F. Zhu W. Avolio B. J. (2006). The impact of efficacy on work attitudes across cultures. *J. World Bus.* 41 121–132. 10.1016/j.jwb.2005.09.003

[B101] Maddux J. E. (1995). “Self-efficacy theory,” in *Self-Efficacy, Adaptation, and Adjustment. The Plenum Series in Social/Clinical Psychology*, ed. Maddux J. E. (Boston, MA: Springer), 3–33. 10.1007/978-1-4419-6868-5_1

[B102] Man T. W. Y. (2019). “Nurturing entrepreneurial competencies through university-based entrepreneurship centers: a social constructivist perspective,” in *Advances in Entrepreneurship, Firm Emergence and Growth*, eds Katz J. A. Corbet A. C. (Bingley: Emerald Publishing Limited), 141–161. 10.1108/S1074-754020190000021006

[B103] Marlow S. McAdam M. (2013). Gender and entrepreneurship. *Int. J. Entrep. Behav. Res.* 19 114–124. 10.1002/(SICI)1097-4636(19970615)35:4<433::AID-JBM3>3.0.CO;2-I

[B104] Mattare M. (2008). *Teaching Entrepreneurship: The Case for an Entrepreneurial Leadership Course. USASBE 2008 Procedeedings.* 78–93. Available online at: http://citeseerx.ist.psu.edu/viewdoc/download?doi=10.1.1.385.5891&rep=rep1&type=pdf (accessed December 13, 2019).

[B105] McGee J. E. Peterson M. (2019). The long-term impact of entrepreneurial self-efficacy and entrepreneurial orientation on venture performance. *J. Small Bus. Manag.* 57 720–737. 10.1111/jsbm.12324

[B106] McKevitt D. Marshall D. (2015). The legitimacy of entrepreneurial mentoring. *Int. J. Entrep. Behav. Res.* 21 263–280. 10.1108/IJEBR-05-2014-0089

[B107] Mitchell M. E. Eby L. T. Ragins B. R. (2015). My mentor, my self: Antecedents and outcomes of perceived similarity in mentoring relationships. *J. Vocat. Behav.* 89 1–9. 10.1016/j.jvb.2015.04.008

[B108] Moore J. H. Wang Z. (2017). Mentoring top leadership promotes organizational innovativeness through psychological safety and is moderated by cognitive adaptability. *Front. Psychol.* 8:318. 10.3389/fpsyg.2017.00318 28303114 PMC5332363

[B109] Nabi G. Liñán F. (2011). Graduate entrepreneurship in the developing world: intentions, education and development. *Educ. Train.* 53 325–334. 10.1108/00400911111147668

[B110] Nabi G. Liñán F. Fayolle A. Krueger N. Walmsley A. (2017). The Impact of entrepreneurship education in higher education: a systematic review and research agenda. *Acad. Manag. Learn. Educ.* 16 277–299. 10.5465/amle.2015.0026

[B111] Nabi G. Walmsley A. Akhtar I. (2019). Mentoring functions and entrepreneur development in the early years of university. *Stud. High. Educ.* 13 1–16. 10.1080/03075079.2019.1665009

[B112] Nathan A. J. Scobell A. (2012). *How China sees America*, 2nd Edn, eds Kenny D. A. Little T. D. (New York, NY: The Guilford Press), 10.1017/CBO9781107415324.004

[B113] Newbery R. Lean J. Moizer J. Haddoud M. (2018). Entrepreneurial identity formation during the initial entrepreneurial experience: The influence of simulation feedback and existing identity. *J. Bus. Res.* 85 51–59. 10.1016/J.JBUSRES.2017.12.013

[B114] Olsson L. E. Gärling T. Ettema D. Friman M. Fujii S. (2013). Happiness and Satisfaction with Work Commute. *Soc. Indic. Res.* 111 255–263. 10.1007/s11205-012-0003-2 23378683 PMC3560964

[B115] O’Mally J. Antonelli K. (2016). The effect of career mentoring on employment outcomes for college students who are legally blind. *J. Vis. Impair. Blind.* 110 295–307. 10.1177/0145482x1611000502

[B116] Oppedal B. L. I. Garcia P. A. J. (2019). Gender, formality, and entrepreneurial success. *Small Bus. Econ.* 19 1–20. 10.1007/s11187-019-00163-8

[B117] Otto K. Rigotti T. Mohr G. (2013). in *The Psychological Effects of Restructuring*, eds Antoniou A. S. G. Cooper C. L. (Cheltenham: Edward Elgar Publishing), 10.4337/9780857933843.00026

[B118] Otto K. Roe R. Sobiraj S. Baluku M. M. Garrido Vásquez M. E. (2017). The impact of career ambition on psychologists’ extrinsic and intrinsic career success: The less they want, the more they get. *Career Dev. Int.* 22 23–36. 10.1108/CDI-06-2016-0093

[B119] Parker S. C. (2009). *The Economics of Entrepreneurship*, 2nd Edn Cambridge: Cambridge University Press, 10.1017/CBO9780511817441

[B120] Patel P. C. Thatcher S. M. B. (2014). Sticking it out: individual attributes and persistence in self-employment. *J. Manage.* 40 1932–1979. 10.1177/0149206312446643

[B121] Podsakoff P. M. MacKenzie S. B. Lee J.-Y. Podsakoff N. P. (2003). Common method biases in behavioral research: a critical review of the literature and recommended remedies. *J. Appl. Psychol.* 88 879–903. 10.1037/0021-9010.88.5.879 14516251

[B122] Politis D. (2005). The process of entrepreneurial learning: A conceptual framework. *Entrep. Theory Pract.* 29 399–424. 10.1111/j.1540-6520.2005.00091.x

[B123] Raabe B. Beehr T. A. (2003). Formal mentoring versus supervisor and coworker relationships: Differences in perceptions and impact. *J. Organ. Behav.* 24 271–293. 10.1002/job.193

[B124] Radu Lefebvre M. Redien-Collot R. (2013). “How to do things with words”: The discursive dimension of experiential learning in entrepreneurial mentoring dyads. *J. Small Bus. Manag.* 51 370–393. 10.1111/jsbm.12022

[B125] Ryan R. M. Deci E. L. (2000). Self-determination theory and the facilitation of intrinsic motivation, social development, and well-being. *Am. Psychol.* 55 68–78. 10.1037//0003-066X.55.1.68 11392867

[B126] Ryan R. M. Deci E. L. (2017). *Self-Determination Theory: Autonomy and Basic Psychological Needs in Human Motivation, Social Development, and Wellness.* New York, NY: The Guilford Press.

[B127] Samman E. (2007). Psychological and Subjective well-being: A proposal for internationally comparable indicators. *Oxford Dev. Stud.* 35 459–486. 10.1080/13600810701701939

[B128] Sánchez J. C. (2013). The impact of an entrepreneurship education program on entrepreneurial competencies and intention. *J. Small Bus. Manag.* 51 447–465. 10.1111/jsbm.12025

[B129] Sappleton N. Lourenço F. (2016). Work satisfaction of the self-employed: The roles of work autonomy, working hours, gender and sector of self-employment. *Int. J. Entrep. Innov.* 17 89–99. 10.1177/1465750316648574

[B130] Sarri K. K. (2011). Mentoring female entrepreneurs: A mentors’ training intervention evaluation. *J. Eur. Ind. Train.* 35 721–741. 10.1108/03090591111160814

[B131] Schummer S. E. Otto K. Hünefeld L. Kottwitz M. U. (2019). The role of need satisfaction for solo self-employed individuals’ vs. employer entrepreneurs’ affective commitment towards their own businesses. *J. Glob. Entrep. Res.* 9:63 10.1186/s40497-019-0190-2

[B132] Scott E. L. Shu P. (2017). Gender gap in high-growth ventures: Evidence from a university venture mentoring program. *Am. Econ. Rev.* 107 308–311. 10.1257/aer.p20171009

[B133] Shir N. Nikolaev B. N. Wincent J. (2018). Entrepreneurship and well-being: the role of psychological autonomy, competence, and relatedness. *J. Bus. Ventur.* 34 1 10.1016/j.jbusvent.2018.05.002

[B134] Skaalvik E. M. Skaalvik S. (2014). Teacher self-efficacy and perceived autonomy: Relations with teacher engagement, job satisfaction, and emotional exhaustion. *Psychol. Rep.* 114 68–77. 10.2466/14.02.PR0.114k14w0 24765710

[B135] Smith B. Tolbert C. M. (2018). Financial motivations and small business longevity: the effects of gender and race. *J. Dev. Entrep.* 23:1850024 10.1142/S1084946718500243

[B136] Stajkovic A. D. Luthans F. (1998). Social cognitive theory and self-efficacy: goin beyond traditional motivational and behavioral approaches. *Organ. Dyn.* 26 62–74. 10.1016/s0090-2616(98)90006-7

[B137] Starr J. A. Fondas N. (1992). A model of entrepreneurial socialization and organization formation. *Entrep. Theory Pract.* 17 67–77. 10.1177/104225879201700108

[B138] St-Jean E. (2012). Mentoring as professional development for novice entrepreneurs: maximizing the learning. *Int. J. Train. Dev.* 16 200–216. 10.1111/j.1468-2419.2012.00404.x

[B139] St-Jean E. Audet J. (2012). The role of mentoring in the learning development of the novice entrepreneur. *Int. Entrep. Manag. J.* 8 119–140. 10.1007/s11365-009-0130-7

[B140] St-Jean E. Audet J. (2013). The effect of mentor intervention style in novice entrepreneur mentoring relationships. *Mentor. Tutoring Partnersh. Learn.* 21 96–119. 10.1080/13611267.2013.784061

[B141] St-Jean E. Radu-Lefebvre M. Mathieu C. (2018). Can less be more? Mentoring functions, learning goal orientation, and novice entrepreneurs’ self-efficacy. *Int. J. Entrep. Behav. Res.* 24 2–21. 10.1108/IJEBR-09-2016-0299

[B142] St-Jean É Mathieu C. (2015). Developing attitudes toward an entrepreneurial career through mentoring. *J. Career Dev.* 42 325–338. 10.1177/0894845314568190

[B143] St-Jean É Tremblay M. (2020). Mentoring for entrepreneurs: a boost or a crutch? Long-term effect of mentoring on self-efficacy. *Int. Small Bus. J. Res. Entrep.* 10.1177/0266242619901058

[B144] Sullivan R. (2000). Entrepreneurial learning and mentoring. *Int. J. Entrep. Behav. Res.* 6 160–175. 10.1108/13552550010346587

[B145] Terjesen S. Sullivan S. E. (2011). The role of developmental relationships in the transition to entrepreneurship: a qualitative study and agenda for future research. *Career Dev. Int.* 16 482–506. 10.1108/13620431111168895

[B146] Ting S. X. Feng L. Qin W. (2017). The effect of entrepreneur mentoring and its determinants in the Chinese context. *Manag. Decis.* 55 1410–1425. 10.1108/MD-07-2016-0477

[B147] Van den Broeck A. Vansteenkiste M. De Witte H. Soenens B. Lens W. (2010). Capturing autonomy, competence, and relatedness at work: construction and initial validation of the work-related basic need satisfaction scale. *J. Occup. Organ. Psychol.* 83 981–1002. 10.1348/096317909X481382 30467716

[B148] Van Emmerik H. (2004). For better and for worse: adverse working conditions and the beneficial effects of mentoring. *Career Dev. Int.* 9 358–373. 10.1108/13620430410526157

[B149] van Gelderen M. (2010). Autonomy as the guiding aim of entrepreneurship education. *Educ. Train.* 52 710–721. 10.1108/00400911011089006

[B150] Wach D. Stephan U. Gorgievski M. (2016). More than money: developing an integrative multi-factorial measure of entrepreneurial success. *Int. Small Bus. J. Res. Entrep.* 34 1098–1121. 10.1177/0266242615608469

[B151] Wanberg C. R. Kammeyer-Mueller J. Marchese M. (2006). Mentor and protégé predictors and outcomes of mentoring in a formal mentoring program. *J. Vocat. Behav.* 69 410–423. 10.1016/j.jvb.2006.05.010

[B152] Wang S. M. Yueh H. P. Wen P. C. (2019). How the new type of entrepreneurship education complements the traditional one in developing entrepreneurial competencies and intention. *Front. Psychol.* 10:2048. 10.3389/fpsyg.2019.02048 31572260 PMC6753869

[B153] Wei X. Liu X. Sha J. (2019). How does the entrepreneurship education influence the students’ innovation? Testing on the multiple mediation model. *Front. Psychol.* 10:1557. 10.3389/fpsyg.2019.01557 31354574 PMC6636545

[B154] Welters R. Mitchell W. Muysken J. (2014). Self determination theory and employed job search. *J. Econ. Psychol.* 44 34–44. 10.1016/j.joep.2014.06.002

[B155] Wieland A. M. Kemmelmeier M. Gupta V. K. McKelvey W. (2019). Gendered cognitions: a socio-cognitive model of how gender affects entrepreneurial preferences. *Entrep. Reg. Dev.* 31 178–197. 10.1080/08985626.2018.1551787

[B156] Wilson F. Kickul J. Marlino D. (2007). Gender, entrepreneurial self-efficacy, and entrepreneurial career intentions: implications for entrepreneurship education. *Entrep. Theory Pract.* 31 387–406. 10.1111/j.1540-6520.2007.00179.x

[B157] Wolf K. Frese M. (2018). Why husbands matter: review of spousal influence on women entrepreneurship in sub-Saharan Africa. *Africa J. Manag.* 4 1–32. 10.1080/23322373.2018.1428019

[B158] Xiao L. North D. (2017). The graduation performance of technology business incubators in China’s three tier cities: the role of incubator funding, technical support, and entrepreneurial mentoring. *J. Technol. Transf.* 42 615–634. 10.1007/s10961-016-9493-4

[B159] Yakın M. Erdil O. (2012). Relationships between self-efficacy and work engagement and the effects on job satisfaction: a survey on certified public accountants. *Procedia - Soc. Behav. Sci.* 58 370–378. 10.1016/j.sbspro.2012.09.1013

[B160] Zapalska A. (1997). International council for small business. *J. Small Bus. Manag.* 10.1111/j.1748-5827.2011.01219.x 22533319

[B161] Zgheib P. (2018). Multi-level framework of push-pull entrepreneurship: comparing American and Lebanese women. *Int. J. Entrep. Behav. Res.* 24 768–786. 10.1108/IJEBR-12-2015-0314

[B162] Zhao X. Lynch J. G. Chen Q. (2010). Reconsidering baron and kenny: myths and truths about mediation analysis. *J. Consum. Res.* 37 197–206. 10.1086/651257

[B163] Zou H. Chen X. Lam L. W. R. Liu X. (2016). Psychological capital and conflict management in the entrepreneur–venture capitalist relationship in China: the entrepreneur perspective. *Int. Small Bus. J. Res. Entrep*. 34 446–467. 10.1177/0266242614563418

